# Cell-to-cell transmission of HSV1 in human keratinocytes in the absence of the major entry receptor, nectin1

**DOI:** 10.1371/journal.ppat.1009631

**Published:** 2021-09-29

**Authors:** Joanne Kite, Tiffany Russell, Juliet Jones, Gillian Elliott

**Affiliations:** Section of Virology, Department of Microbial Sciences, School of Biosciences and Medicine, University of Surrey, Guildford, United Kingdom; Washington State University, UNITED STATES

## Abstract

Herpes simplex virus 1 (HSV1) infects the stratified epithelia of the epidermis, oral or genital mucosa, where the main cell type is the keratinocyte. Here we have used nTERT human keratinocytes to generate a CRISPR-Cas9 knockout (KO) of the primary candidate HSV1 receptor, nectin1, resulting in a cell line that is refractory to HSV1 entry. Nonetheless, a small population of KO cells was able to support infection which was not blocked by a nectin1 antibody and hence was not a consequence of residual nectin1 expression. Strikingly at later times, the population of cells originally resistant to HSV1 infection had also become infected. Appearance of this later population was blocked by inhibition of virus genome replication, or infection with a ΔUL34 virus defective in capsid export to the cytoplasm. Moreover, newly formed GFP-tagged capsids were detected in cells surrounding the initial infected cell, suggesting that virus was spreading following replication in the original susceptible cells. Additional siRNA depletion of the second major HSV1 receptor HVEM, or PTP1B, a cellular factor shown elsewhere to be involved in cell-to-cell transmission, had no effect on virus spread in the absence of nectin1. Neutralizing human serum also failed to block virus transmission in nectin1 KO cells, which was dependent on the receptor binding protein glycoprotein D and the cell-to-cell spread glycoproteins gI and gE, indicating that virus was spreading by direct cell-to-cell transmission. In line with these results, both HSV1 and HSV2 formed plaques on nectin1 KO cells, albeit at a reduced titre, confirming that once the original cell population was infected, the virus could spread into all other cells in the monolayer. We conclude that although nectin1 is required for extracellular entry in to the majority of human keratinocytes, it is dispensable for direct cell-to-cell transmission.

## Author summary

Herpes simplex virus 1 (HSV1) infects the epithelia of the epidermis, oral or genital mucosa to cause cold sores, genital herpes, or more serious outcomes such as keratitis and neonatal herpes. Like many viruses, HSV1 can spread through the extracellular environment or by direct cell-to-cell transmission, with the latter mechanism being important for avoiding antibody responses in the host. Here we have studied HSV1 entry and transmission in the human keratinocyte, the main cell type in the target epithelia, by generating a CRISPR-Cas9 knockout of the primary candidate virus receptor, nectin1. While HSV1 was unable to infect the majority of nectin1 knockout keratinocytes, a small population of these nectin1 KO cells remained susceptible to virus entry, and once infected, the virus was able to spread into the rest of the monolayer. This spread continued in the presence of neutralising serum which blocks extracellular virus, and required glycoprotein D, the main virus receptor-binding protein, and glycoproteins gE and gI which are known to be involved in cell-to-cell spread. Hence, while nectin1 is required for virus entry into the majority of human keratinocyte cells, it is dispensable for cell-to-cell transmission of the virus. These data have implications for the mechanism of HSV1 epithelial spread and pathogenesis.

## Introduction

Herpes simplex virus type 1 (HSV1) infects and is generally restricted to the stratified epithelial cells of the skin, oral mucosa, cornea or the genital mucosa before transmitting to and establishing lifelong latent infection in sensory neurons [1]. It reactivates periodically to re-enter the infectious cycle, when it travels back to the epithelia at the initial site of infection and replicates to cause either the cold sore, ocular herpes or genital herpes. Given that the major cell type of these epithelia is the keratinocyte, the molecular events involved in HSV entry and transmission in these cells are highly relevant to understanding the biology of the virus. The most frequently used keratinocyte cell line in HSV1 studies to date has been the HaCaT cell line, a spontaneously immortalised aneuploid line that is unable to undergo terminal epidermal differentiation [2]. By contrast, we have been studying HSV1 entry in the nTERT keratinocyte line, a diploid line derived from primary human keratinocytes. These cells have been retrovirally induced to express the catalytic subunit of the telomerase holoenzyme hTERT, and have spontaneously lost the function of p16^INK4a^ which usually inhibits the transition from G1 to S phase, allowing the maintenance of growth for much longer than normal primary cell types [3]. Crucially, nTERT cells are still dependent on epidermal growth factor and are able to differentiate [4]. Hence, these cells offer a tractable system that are physiologically relevant for the investigation of HSV1 infection of human tissue. Of note, HSV1 enters and replicates rapidly in these human keratinocytes [5], indicating that the virus is exquisitely adapted to grow in these cells.

HSV1 entry involves four essential virus-encoded envelope proteins–the receptor binding protein glycoprotein D (gD), and the core fusion machinery comprising glycoproteins gB, gH and gL [6]. Two major cell receptors have been identified for gD—HveC, more commonly known as the adhesion molecule nectin1 [7], and HVEM (HveA), the first HSV-1 receptor identified, and a member of the tumour necrosis factor receptor family [8] which is expressed predominantly on lymphoid cells [9]. A number of other receptors have been identified, including a modified form of heparan sulfate on cell surface proteoglycans, 3-O-sulfated heparin sulfate [10], nectin2 (HveB) for HSV2 gD [11], and the gB receptor paired immunoglobulin receptor alpha, PILRα, which is also expressed on immune cells [12]. Nectin1 is expressed in both keratinocytes and neurons [7] and has been shown to be highly expressed in HaCaT cells [13]. As such, our previous siRNA depletion studies identifying nectin1 as the preferred receptor over HVEM for HSV1 entry into human nTERT keratinocytes as well as HeLa cells [5], was in broad agreement with other work on human and murine keratinocytes [14]. HSV1 entry is generally studied using cell-free virus infection of monolayers, but cell-to-cell spread of HSV1, whereby virus transmits directly from already infected cells [15], is proposed to be the main route of spread in infected human tissue [16]. Nonetheless, the molecular mechanism of direct cell-to-cell transmission of HSV1 is poorly characterised. The four entry proteins gD, gB, gH and gL are sufficient for infection from the extracellular environment, but efficient infection by cell-to-cell transfer additionally requires glycoproteins gE and gI [17], which are non-essential in tissue culture, but essential for *in vivo* infection [18] adding further weight to the importance of cell-to-cell spread in the host. Although one study has reported that nectin1 is required for cell-to-cell spread in a number of cell types, keratinocytes were not included in that study [19]. Moreover, it was recently suggested that inhibition of the ER-bound protein tyrosine phosphatase 1B (PTP1B) specifically blocks cell-to-cell spread of HSV1, suggesting that tyrosine phosphorylation regulates either the cellular or viral trafficking proteins that make up the cell-to-cell spread machinery [20].

In this current study we wished to generate a tractable tool for HSV1 entry studies by using CRISPR-Cas9 to knock out nectin1 expression in nTERT keratinocytes. As anticipated from previous studies, this resulted in a cell line that was broadly resistant to HSV1 infection, and which lost that resistance when stably transfected to express nectin1 tagged with a V5 epitope. Nonetheless, a small population of nectin1 knockout cells retained susceptibility to HSV1 infection. Intriguingly, these cells supported virus replication, with progeny virus spreading unhindered in the nectin1 KO monolayers, allowing for the production of plaques–a property shared with HSV2. This transmission required gD, but not HVEM or PTP1B, and was not blocked by the presence of neutralising human serum suggesting that it occurred by direct cell-to-cell spread. Nonetheless, ΔgI or ΔgE variants of HSV1, which infected the same proportion of KO cells as Wt virus, were equally defective for virus spread on KO cells and nTERT cells, indicating that this complex was still involved in transmission in the absence of nectin1. Hence, we have discovered that the gI- gE- dependent cell-to-cell transmission pathway of HSV1, which is important for virus spread in the host, can function independently of nectin1, the major HSV1 entry receptor in human keratinocytes.

## Results

### Human keratinocyte cells express high levels of the nectin1 receptor

**Fig 1:**
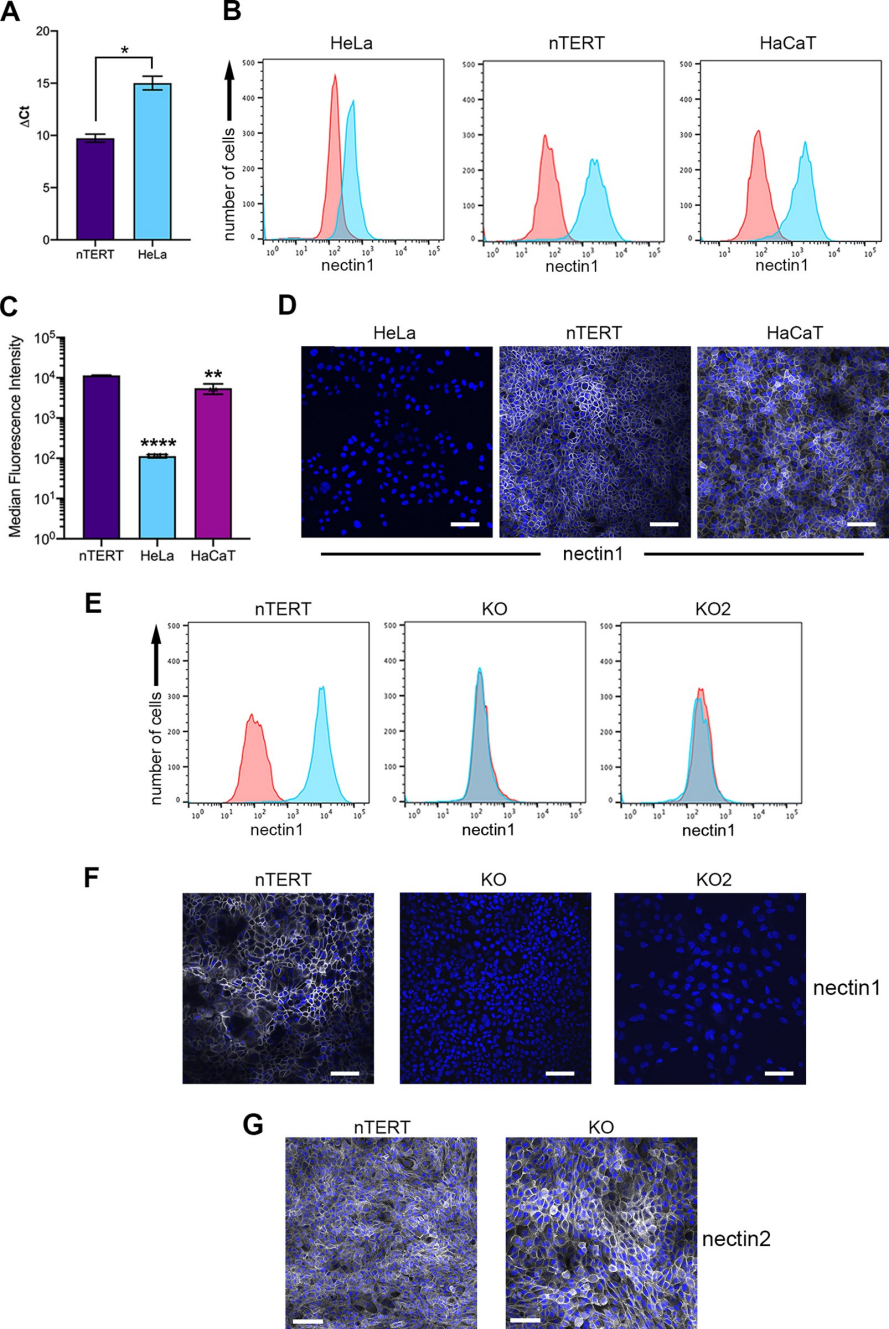
Human keratinocytes express high levels of the major HSV1 receptor, nectin1. **(A)** Total RNA was isolated from HeLa and nTERT cells, and subjected to RT-qPCR using primers for nectin1. Results are presented as ΔCt measurement using 18s as reference (mean±SEM, *n* = 3). **(B)** HeLa, nTERT and HaCaT cells were fixed and stained for nectin1 (blue) or secondary alone (red) before being analysed by flow cytometry. **(C)** Median fluorescence intensity of cell surface nectin1 staining in nTERT, HeLa and HaCaT cells (mean±SEM, *n* = 3). **(D)** HeLa, nTERT and HaCaT cells were grown on coverslips before fixation and staining of non-permeabilised cells for nectin1. Scale bar = 100 μm. **(E)** nTERT, KO and KO2 cells were stained and fixed for nectin1 (blue) or secondary alone (red) before being analysed by flow cytometry. **(F)** nTERT, KO and KO2 cells were grown on coverslips before fixation and staining of non-permeabilised cells for nectin1. **(G)** As for (**F)**, but cells were stained for nectin2. Scale bar = 100 μm. *, P < 0.05; **, P < 0.01; ****, P < 0.0001 (by one-way analysis of variance [ANOVA]).

HSV1 infects keratinocyte cells in the human. It is therefore noteworthy that our previous work has shown that nTERT keratinocyte cells express around 60-fold more nectin1 mRNA than HeLa cells [5]. Moreover, the spontaneously transformed immortal keratinocyte HaCaT cell line also expresses high levels of cell surface nectin1 protein [13]. In our previous work, we did not have suitable tools available to measure endogenous nectin1 protein levels, but assuming this mRNA level correlates with a protein level similar to HaCaT cells, it would suggest that the natural target cell for the virus is generally extremely rich in its major receptor. In turn, this could explain our previous data demonstrating rapid entry into keratinocytes [5]. Having now acquired an antibody which recognises human nectin1 by flow cytometry, and to a lesser extent immunofluorescence, we have extended this data to determine the level of nectin1 protein on the cell surface of relevant cell types. RT-qPCR confirmed the high level of nectin1 mRNA in nTERT cells compared to HeLa cells (Fig 1A). Flow cytometry on non-permeabilised cells stained for cell-surface nectin1 revealed that nTERT and HaCaT keratinocytes express around 50-fold and 20-fold more nectin1 respectively on their cell surface compared to HeLa cells (Fig 1B and 1C). When the same antibody was used to stain the surface of each of these cell types for analysis by confocal microscopy, nectin1 was readily detectable on nTERT and HaCaT cells but not on HeLa cells (Fig 1D) confirming that high level cell surface expression of the HSV1 nectin1 receptor is a feature of human keratinocytes. As all these cell types support HSV1 entry, and our previous data suggests that at least in HeLa cells this is via nectin1 [5], we surmise that there is sufficient nectin1 receptor on HeLa cells to support virus entry, but that our previous determination of rapid entry into keratinocyte cells is due at least in part to this much higher availability of the nectin1 receptor for HSV1 [5].

### Generation of a CRISPR-Cas9 nectin1 knockout in nTERT human keratinocytes

To further investigate the contribution of nectin1 to HSV1 entry into keratinocyte cells, and to establish a tractable tool for entry studies, we next generated an nTERT nectin1 knockout cell line using CRISPR-Cas9 mediated gene editing, focusing our efforts on the diploid nTERT cell type rather than the aneuploid HaCaT cell line. nTERT cells transfected with plasmids expressing Cas9 and *NECTIN-1* gRNAs targeting the 5’ end of the gene were selected by growth in puromycin, followed by clonal isolation. Candidate nectin1 knockout (KO) cells were cell-surface stained and analysed by flow cytometry and confocal microscopy to determine nectin1 expression on these cells, with only results of the cell line selected for future work being shown here (KO, Fig 1E and 1F). This confirmed that in comparison to nTERT cells these KO cells did not express nectin1 to a level detectable by either flow cytometry or confocal microscopy. Subsequent sequencing of the nectin1 gene revealed that these KO cells were a mixed population containing a number of CRISPR-Cas9 induced edits in the sequences around the gRNA site (S1 Fig) but importantly no parental sequence was present. Due to the nature of their selection, these cells were found to constitutively express Cas9 (S2 Fig), providing a possible explanation for the presence of multiple changes in the nectin1 sequence. Although the phenotype of these cells was clearly nectin1 knockout, we also additionally isolated a partner CRISPR-Cas9 edited cell-line through single cell sorting of transfected cells 72 hours after transfection rather than by puromycin selection, to produce cells lacking both nectin1 and Cas9 expression (KO2, Fig 1E and 1F). In studies presented below, we found no difference in the behaviour of the Cas9 expressing and non-expressing nectin1 knockout cells. Moreover, these KO cells were found to express a similar level of nectin2 compared to parental nTERT cells (Fig 1G), providing us with a means of separating the relative contribution of these two nectin molecules in HSV entry.

### Characterisation of HSV1 infection in nectin1 KO nTERT cells

**Fig 2:**
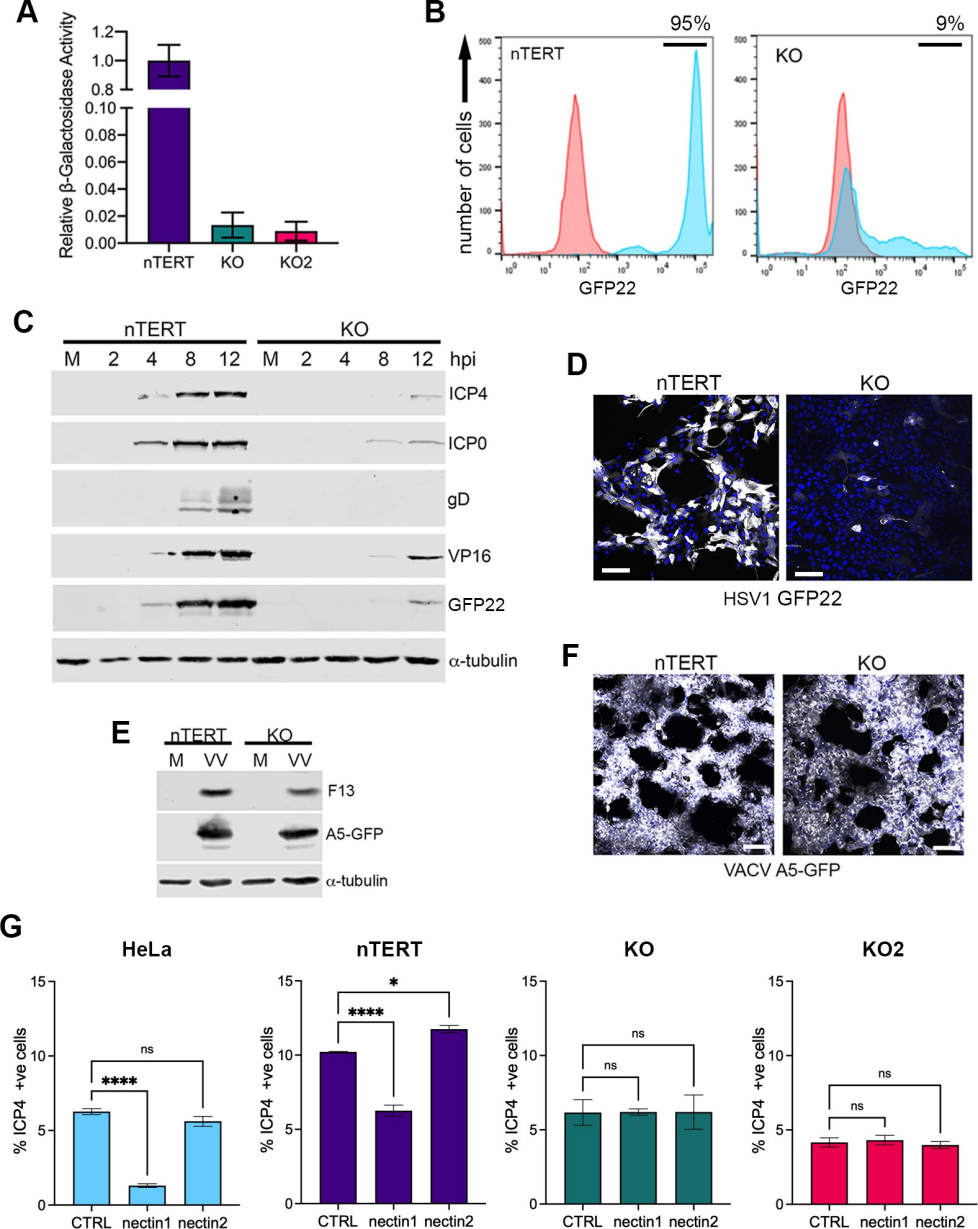
HSV1 infection of nectin1 KO cells. **(A)** nTERT, KO and KO2 cells were infected at MOI 5 with HSV1 IE110*lacZ* on ice to allow attachment, before shifting to 37°C for 3 h, and β-gal activity was measured by ONPG assay (mean±SEM, *n* = 3). **(B)** nTERT and KO cells were infected with HSV1 expressing GFP22 at MOI 5, and fixed 8 h later. Uninfected (red) and infected (blue) cells were analysed for GFP fluorescence by flow cytometry. Percentages refer to proportion of cells with GFP > 10^4^ fluorescence intensity. **(C)** nTERT and KO cells were infected with HSV1 GFP22 at MOI 5, harvested at the indicated times and analysed by SDS-PAGE and Western blotting for a range of virus proteins as indicated, and α-tubulin as a loading control. **(D)** nTERT and KO cells grown on coverslips were infected with HSV1 GFP22 at MOI 5, fixed at 8 h, stained with DAPI (blue) and analysed by confocal microscopy for GFP fluorescence (white). Scale bar = 100 μm. **(E)** nTERT and KO cells were infected with VACV expressing A5GFP at MOI 5, harvested at 16 h and analysed by SDS-PAGE and Western blotting for F13 and GFP. **(F)** nTERT and KO cells grown on coverslips were infected with VACV as in (E), fixed, stained with DAPI (blue) and analysed by confocal microscopy for GFP fluorescence (white). Scale bar = 100 μm. **(G)** HeLa, nTERT, KO or KO2 cells were pre-incubated with no antibody (CTRL) or 10 μg nectin1 or nectin2 antibody. Cells were then infected with HSV1 GFP22, acid washed, fixed at 4 h and stained with DAPI. GFP positive cells were counted and expressed as a percentage of total DAPI counts (mean±SEM, *n* = 3). *, P < 0.05; ****, P < 0.0001; ns, non-significant (by one-way analysis of variance [ANOVA]).

For preliminary analysis of the ability of these nectin1 KO cells to support HSV1 entry, cells were synchronously infected with HSV1 expressing *β-galactosidase* under the control of the immediate early (IE) ICP0 promoter [21], and after 3 h, *β-galactosidase* activity was measured as a surrogate for virus entry [22] (Fig 2A). In contrast to nTERT cells, where *β-galactosidase* activity was high, both nectin1 KO lines expressed greatly reduced levels of *β-galactosidase*, suggesting that early events in HSV1 infection had indeed been abrogated.

To look in more detail at HSV1 infection in the absence of nectin1, KO cells were infected with HSV1 expressing the virus protein VP22 as a GFP fusion protein (GFP22), and analysed by flow cytometry for GFP at 8 h in comparison to infected nTERT cells. These results broadly confirmed the entry assay, indicating a massive reduction in GFP22 positive cells in the absence of nectin1 (Fig 2B). Nonetheless, Western blotting of a high multiplicity time-course of infection further indicated that KO cells were able to support low level virus protein expression, but none of the proteins tested, including immediate-early proteins ICP4 and ICP0 were detectable much before 12 h, compared to nTERT cells where they were readily detectable by 4 h (Fig 2C). Imaging of GFP22 fluorescence in nTERT and KO cells infected in the same way revealed that these low protein levels were a consequence of HSV1 infecting a small number of KO compared to nTERT cells (Fig 2D). By contrast, high multiplicity infection of nTERT and KO cells with vaccinia virus (VACV) expressing A5-GFP revealed that VACV protein expression was unaffected by the absence of nectin1 (Fig 2E), a result that was corroborated by fluorescence microscopy of VACV infected cells where all nTERT and KO cells were shown to be A5-GFP positive (Fig 2F). Taken together, these data indicate that the majority of KO cells are refractory specifically to HSV1 infection, but that a small subpopulation retain the ability to support infection.

To address the possibility that, despite CRISPR-Cas9 knockout of the nectin1 gene, a residual level of nectin1 expression remained in the knockout cells, the ability of the nectin1 antibody to block entry into the susceptible population of KO cells was tested. HeLa, nTERT, KO and KO2 cells were all incubated on ice in the presence of no antibody, or 10 μg of either nectin1 or nectin2 antibody. Following infection with the GFP22 virus at an MOI required to infect approximately 5 to 10% of cells, the cells were fixed at 4 h and GFP positive cells counted. Pre-incubation with the nectin1 antibody but not the nectin2 antibody resulted in a significant reduction in the number of infected HeLa and nTERT cells (Fig 2G). Although the reduction was not as great in nTERT cells, this is likely due to the high level of nectin1 present on the surface of these cells, as seen in work from others [23]. Nonetheless, incubation of KO and KO2 cells with the nectin1 antibody had no effect on the ability of HSV1 to enter these cells, indicating that this entry was not via a low-level expression of nectin1 (Fig 2G). Moreover, incubation with the nectin2 antibody also had no effect on entry into nectin1 KO or KO2 cells (Fig 2G), and hence the virus does not gain access to these cells via an alternative interaction with the nectin2 receptor.

**Fig 3:**
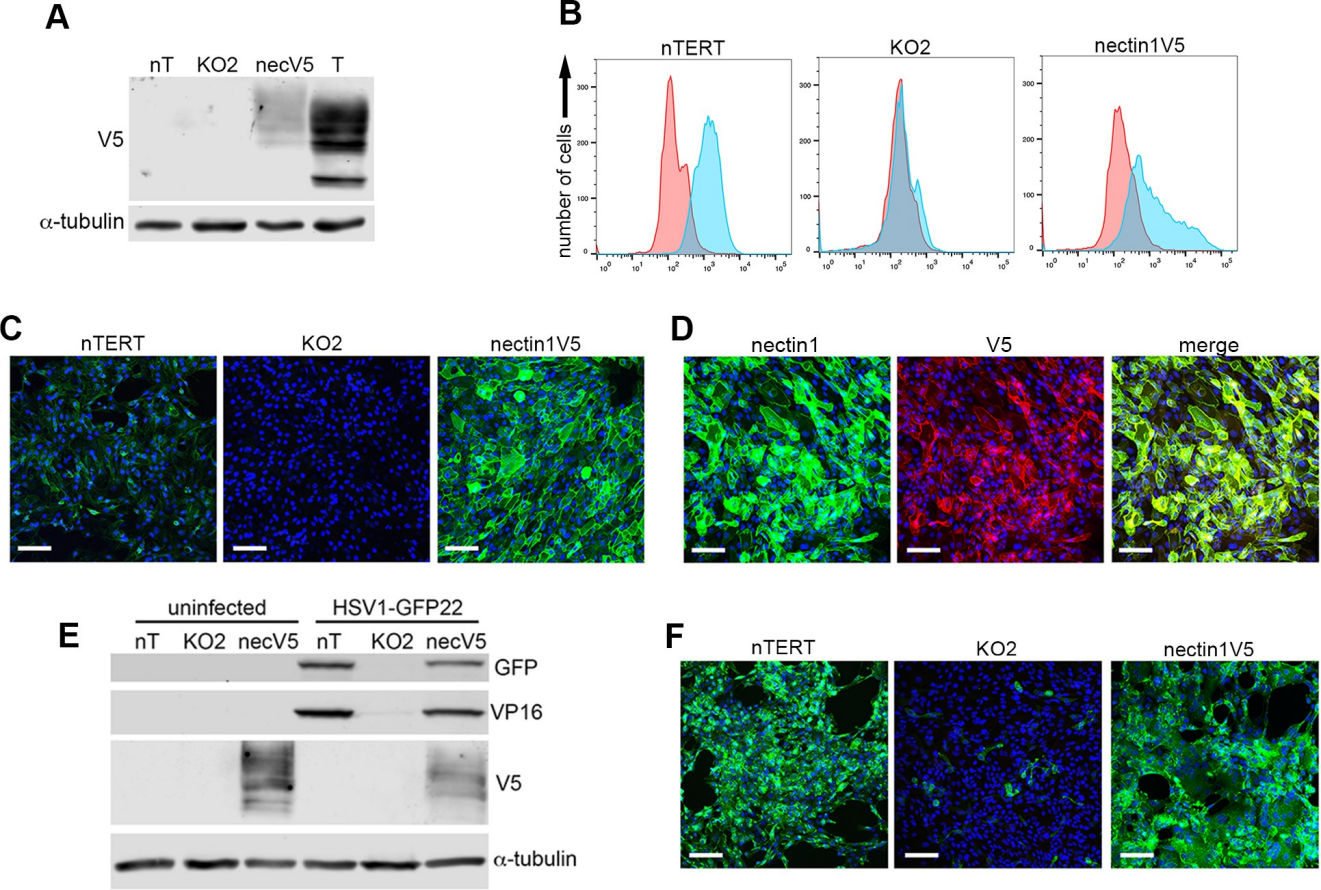
HSV1 infection is rescued in nectin1 knockout cells stably expressing nectin1V5. **(A)** KO2 cells that had been selected for stable nectin1V5 expression (necV5) were analysed by SDS-PAGE and Western blotting with antibody for V5 and α-tubulin alongside nTERT (nT) and KO2 cells. T, nTERT cells transiently transfected with plasmid expressing nectin1V5. **(B)** Flow cytometry of non-permeabilised nTERT, KO and nectinV5 cells was carried out using nectin1 antibody (blue) compared to secondary only (red) to measure nectin1V5 at the cell surface. **(C)** nTERT, KO2 and nectin-V5 cells were grown on coverslips, fixed without permeabilisation and stained with nectin1 antibody (green). **(D)** Nectin1V5 cells grown on coverslips were fixed, permeabilised and stained with antibodies to nectin1 (green) and V5 (red). Scale bar = 100 μm. **(E)** nTERT, KO and nectin1V5 cells were infected with HSV1 expressing GFP22 MOI 5 and analysed by SDS-PAGE and Western blotting after 12 h for GFP, VP16, V5 and α-tubulin. **(F)** As for (E) but cells were grown on coverslips, and infected cells were fixed at 8 h nuclei stained with DAPI (blue) and cells imaged for GFP fluorescence (green). Scale bar = 100 μm.

As final confirmation that the phenotype of these cells was a consequence of nectin1 knockout, the KO2 cells were engineered to stably express nectin1 tagged at its C-terminus with the V5 epitope. Appropriate expression of nectin1V5 was first confirmed by transient transfection of nTERT and HeLa cells followed by Western blotting for V5 (S3 Fig). This showed a V5-tagged protein which was expressed as multiple forms, which were further shown to be a consequence of *N-*linked glycosylation by treatment with the deglycosylation enzyme PNGaseF (S3 Fig). Immunofluorescence of nectin1V5 expressing nTERT cells also confirmed that V5-tagged nectin1 localised to the plasma membrane (S3 Fig). Following clonal selection of a KO2 line that had been stably transfected with the nectin1V5 plasmid, cells were tested for nectin1V5 expression by Western blotting, which indicated expression of a V5-tagged protein of the appropriate molecular weight and of similar glycosylation pattern to that seen in transiently transfected cells (Fig 3A). Flow cytometry and immunofluorescence for cell-surface nectin1 indicated that these cells expressed high levels of nectin1 on the plasma membrane in comparison to nTERT cells, albeit at levels that varied from cell to cell (Fig 3B and 3C). Imaging of permeabilised cells stained with antibodies for nectin1 and V5, which should be cell surface and cytoplasmic respectively, confirmed correct localisation of nectin1V5 (Fig 3D). Moreover, Western blotting of nectin1V5 cells infected with HSV1 expressing GFP22 indicated that in comparison to KO2 cells, these cells had regained the ability to support infection to a level similar to nTERT cells (Fig 3E), a result that was confirmed by GFP fluorescence which in contrast to KO2 cells, was present in the full population of nectinV5 cells (Fig 3F).

### nTERT keratinocytes support ongoing HSV1 infection in the absence of nectin1

**Fig 4:**
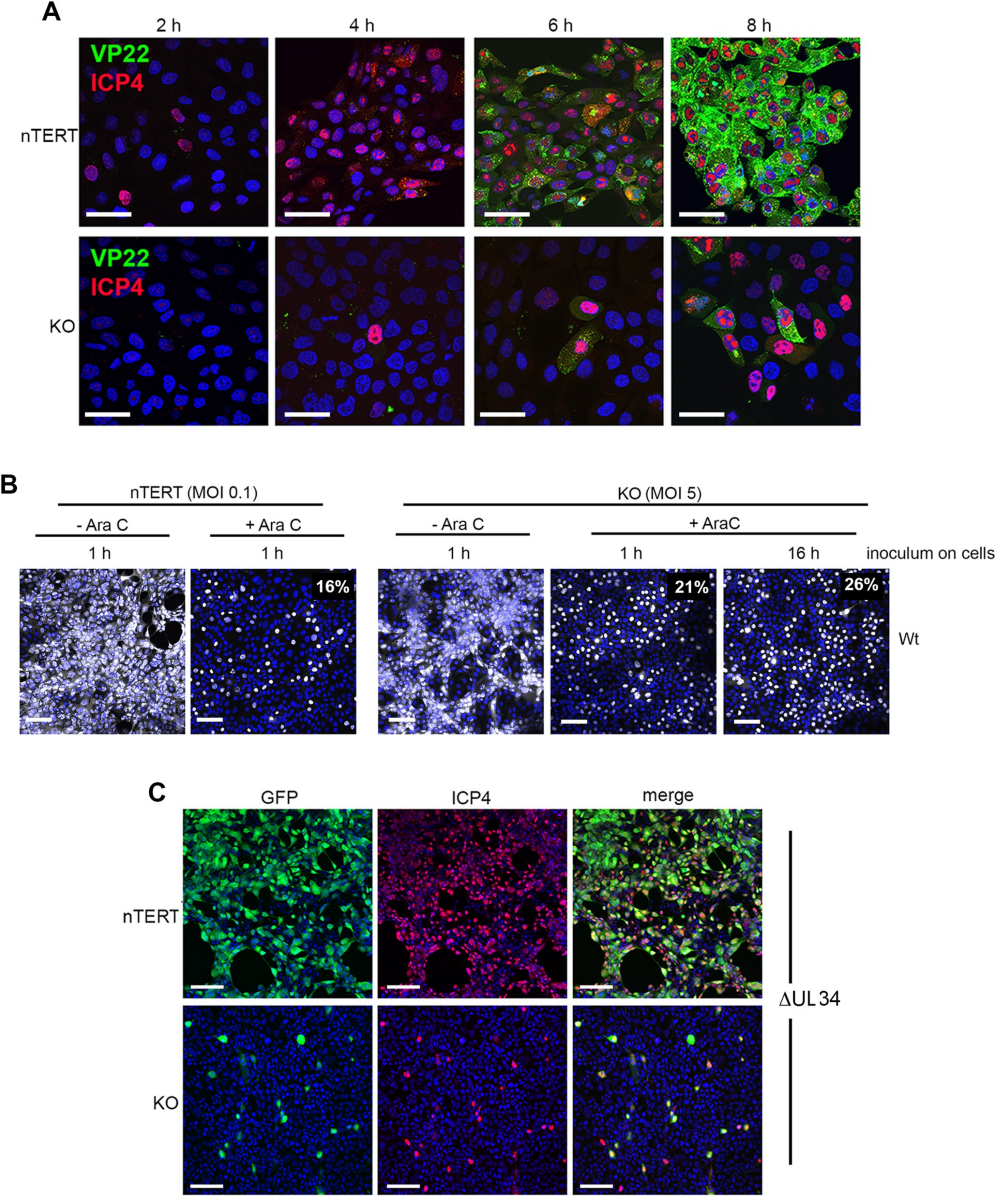
HSV1 exhibits delayed infection in majority of nectin1 KO cells. **(A)** nTERT and KO cells grown on coverslips were infected with HSV1 GFP22 (green) at MOI 5 and fixed at the indicated times. Cells were permeabilised and stained for ICP4 (red) and nuclei were stained with DAPI (blue). Scale bar = 50 μm. **(B)** nTERT and KO cells were infected with HSV1 Sc16 at MOI 0.1 or 5 respectively (as determined in Vero cells) in the absence or presence of 100 ng/ml AraC for the whole experiment. Inoculum was removed after one hour as normal (1 h), or left on for the course of the infection (16 h). Cells were fixed at 16 h and stained for ICP4 (white) and nuclei were stained with DAPI (blue). Scale bar = 100 μm. **(C)** nTERT and KO cells grown on coverslips were infected with HSV1 ΔUL34 at MOI 5 and fixed at 20 h. Cells were permeabilised and stained for ICP4 (red) and nuclei were stained with DAPI (blue). GFP expressed in place of UL34 is in green. Scale bar = 100 μm. Percentages refer to number of ICP4 positive nuclei, determined using NIH ImageJ software.

Closer examination of the infected cell profiles shown in Fig 2B revealed that a small number of KO cells expressed high levels of GFP22 compared to 95% of nTERT cells, but that a similar population of cells also expressed a low level of GFP22. To examine this at the single cell level, a time course of nTERT and KO cells infected at high multiplicity with HSV1 expressing GFP22 was stained for ICP4, which exhibits a transition in localisation according to the time of infection: initially in a diffuse nuclear pattern, followed by localisation to nuclear replication compartments, and finally at later times in a cytoplasmic punctate pattern; it can therefore be used to monitor the kinetic stage of HSV1 infection. This revealed that the majority of nTERT cells expressed ICP4 by 4 h and GFP22 by 6 h (Fig 4A), in line with the known replication kinetics of HSV1 in nTERT keratinocytes [5]. By contrast, only a small number of KO cells expressed ICP4 at 4 h and a similar number expressed GFP22 by 6 h (Fig 4A, KO). Nonetheless, by 8 h, many more cells were positive for ICP4 in its early diffuse nuclear localisation pattern, without expressing GFP22, suggesting these cells were at an early stage of infection (Fig 4A, KO 8 h). Taken together, the flow cytometry and microscopy results suggest that in the absence of nectin1, a small number of nTERT cells are infected with HSV1 with similar kinetics to nectin1-expressing nTERT cells, while a second population exhibits a delay in the onset of immediate-early gene expression.

As the virus inoculum had not been inactivated in this experiment, this pattern of delayed ICP4 expression in nectin1 KO cells could be explained either by delayed infection from the initial virus inoculum or the spread of newly replicated virus from the initially infected cells into a larger population of uninfected cells. To determine if a new round of genome replication was required, nTERT and KO cells were infected with Wt virus in the absence or presence of the DNA replication inhibitor AraC, fixed at 16 h and stained for ICP4, indicating that AraC blocked the increase in the number of infected cells in both cell types (Fig 4B, compare -AraC and + AraC). Moreover, prolonged exposure of KO cells to the HSV1 inoculum in the presence of AraC had little effect on the number of initially infected cells, indicating that the failure to infect a larger proportion of cells in the first instance was not simply a consequence of a reduced rate of attachment/entry (Fig 4B, KO +AraC, compare percentage of ICP4 positive cells at 1h and 16h). To confirm that newly produced virus was required for the observed delay in infection, we made use of a virus expressing GFP in place of the nuclear egress protein UL34 (ΔUL34). This deletion mutant expresses all proteins including late proteins as normal, and capsids assemble in the nucleus, but they fail to translocate to the cytoplasm for subsequent envelopment [24]. In this case, while all nTERT cells expressed both ICP4 and GFP at this late time when infected at high multiplicity (Fig 4C, nTERT), only the original susceptible subpopulation of KO cells expressed these proteins (Fig 4C, KO), indicating that ICP4 expression in the later population of cells required the production of infectious virions.

### HSV1 spreads to neighbouring nectin1 KO cells

**Fig 5:**
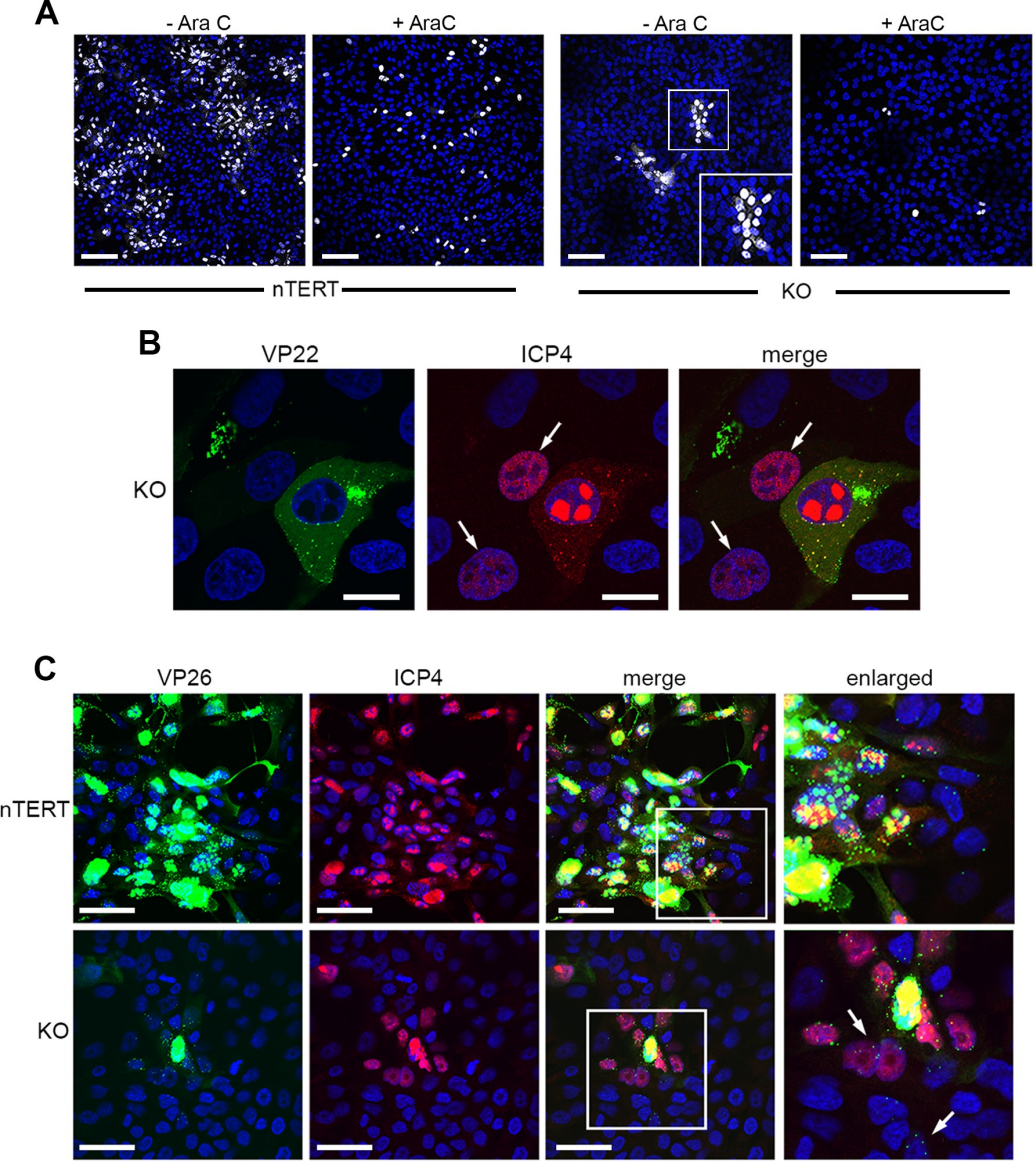
HSV1 infection spreads in nectin 1 KO cells. **(A)** nTERT and KO cells were infected with HSV1 Sc16 at MOI 0.05 in the absence or presence of 100 ng/ml AraC to block virus genome replication. Cells were fixed at 12 h and stained for ICP4 (white) and nuclei were stained with DAPI (blue). Scale bar = 100 μm. **(B)** nTERT and KO cells grown on coverslips were infected with HSV1 GFP22 (green) at MOI 5 and fixed at 8 h. Cells were permeabilised and stained for ICP4 (red) and nuclei were stained with DAPI (blue). Scale bar = 20 μm. White arrows indicate cells with recent expression of ICP4. **(C)** nTERT and KO cells grown on coverslips were infected with HSV1 GFP-VP26 at MOI 5 and fixed at 12 h. Cells were permeabilised and stained for ICP4 (red) and nuclei were stained with DAPI (blue). GFP-VP26 is in green. Scale bar = 50 μm. Arrow points to cells with GFP-tagged capsids next to the original infected cell.

The above results indicate that virus infectivity is able to spread through nectin1 KO cells. This was unexpected because the initial susceptibility of these cells was around 5 to 10%. To assess how this new round of infection was transmitted through the monolayer, conditions for detecting virus spread through the cells were first established. In this case, low multiplicity infections of nTERT cells in the absence and presence of AraC were fixed at different times after infection and stained for ICP4 by immunofluorescence (S4 Fig). This indicated that under these conditions spread was detectable at 12 hours, and that as expected from the fully susceptible nature of these cells, this spread occurred in the immediate locality of the initially infected cell, spreading in to all cells over 16 h (S4 Fig). Using the same conditions on KO cells, we were also able to detect local infection around the initial infected cell which was absent in AraC treated cells (Fig 5A). Moreover, high magnification imaging of KO cells infected with HSV1 expressing GFP22 and stained for ICP4 revealed that cells containing a low level of ICP4 in the nucleus were frequently located beside cells expressing high level of ICP4 and GFP22 (Fig 5B, arrowed). Additional imaging of cells infected with HSV1 expressing GFP-26 also allowed the visualisation of virus capsid localisation in ICP4-positive nectin1 KO cells (Fig 5C). In both nTERT and KO cells, individual progeny capsids were clearly detectable not only in the initially infected cells, but also in cells surrounding the first infected cell, some of which were already producing ICP4 (Fig 5C, arrowed). These results confirm that while more than 90% of the nectin1 KO cells are resistant to HSV1 infection from virions in the medium, they are fully susceptible to infection from virus transmitting from adjacent cells.

**Fig 6:**
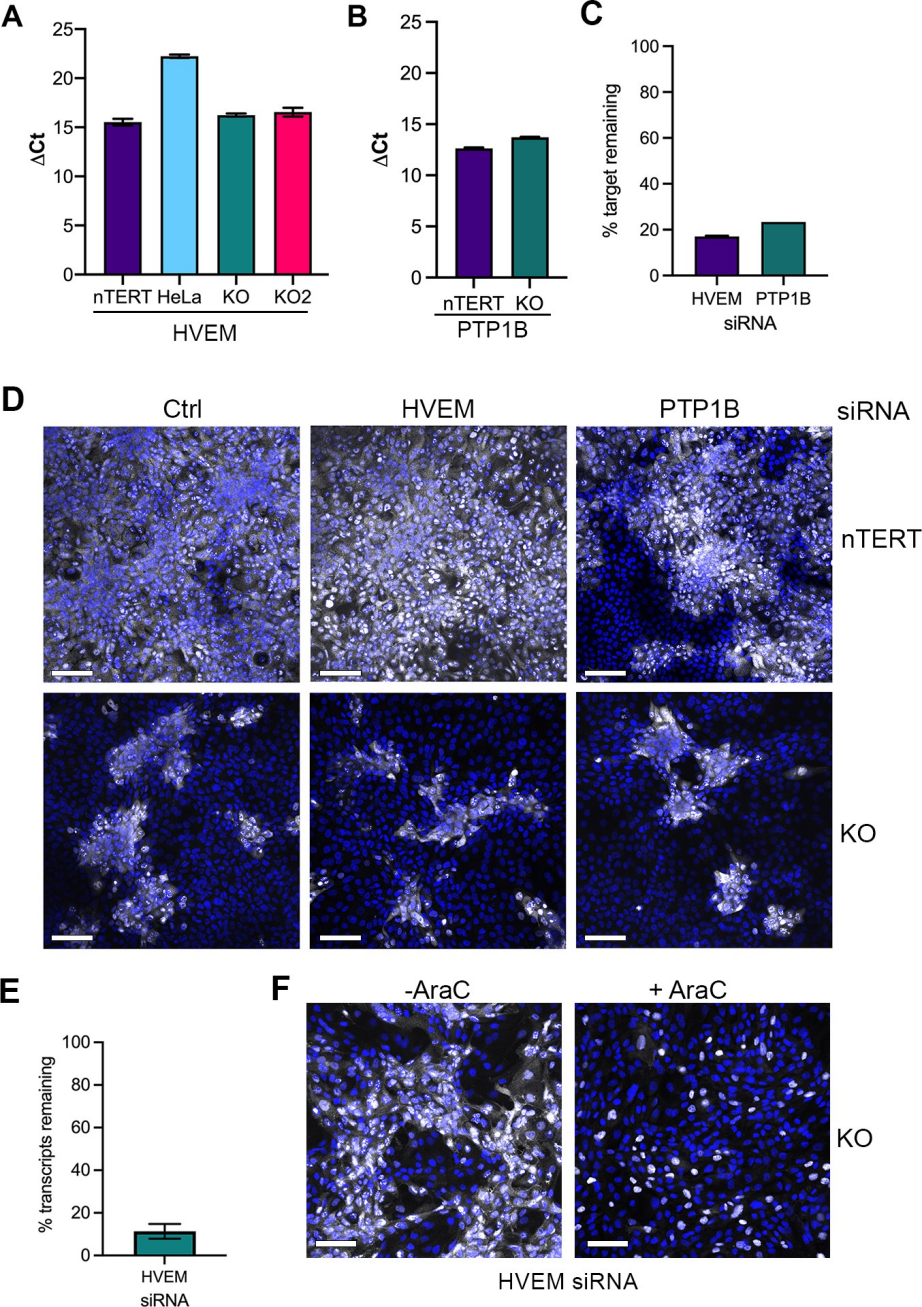
HSV1 spread in the absence of nectin1 does not require HVEM or PTP1B. **(A)** Total RNA was isolated from HeLa, nTERT, KO and KO2 cells, and subjected to RT-qPCR using primers for HVEM. Results are presented as ΔCt measurement using 18s as reference (mean±SEM, *n* = 3). **(B)** Total RNA was isolated from nTERT and KO cells and subjected to RT-qPCR using primers for PTP1B. Results are presented as ΔCt measurement using 18s as reference (mean±SEM, *n* = 3). **(C)** KO cells were transfected with control, HVEM or PTP1B siRNA, and total RNA harvested after 48 h and RT-qPCR performed. Level of mRNA in siRNA transfected cells is presented as the percentage of mRNA in control siRNA transfected cells. **(D)** nTERT and nectin1 KO cells transfected at the same time as in **(C)** were infected after 48 h with HSV1 Sc16 MOI 0.1 or 5 respectively, fixed at 16 h and stained for ICP4 (white). Nuclei were stained with DAPI (blue). **(E)** and **(F)** KO cells were transfected with HVEM siRNA and after 48 h were (E) subjected to RT-qPCR, or (F) infected with HSV1 Sc16 MOI 5 in the presence or absence of 100 ng/ml AraC for the course of the experiment, and fixed and stained for ICP4 (white) at 16 h. Scale bar = 100 μm.

Although our previous data had suggested that HVEM has little role in entry into nTERT keratinocytes when nectin1 is present [5], the above results led us to investigate if depletion of HVEM in the nectin1 KO cells affected virus entry and/or spread in the absence of nectin1. Additionally, we wanted to determine if the previously identified spread factor PTP1B [20] played a role in the nectin1-independent spread of HSV1 in keratinocytes. First, we determined that that HVEM and PTP1B transcripts are expressed at similar levels in both nTERT and KO cells (Fig 6A and 6B). Next, nTERT and KO cells were transfected with control, HVEM or PTP1B siRNAs, and RT-qPCR was used to confirm efficient depletion of each transcript (Fig 6C). Subsequent infection with Wt virus, followed by fixation at 16 h and staining for ICP4 showed that spread of HSV1 in nTERT or KO cells was not altered in cells depleted of HVEM or PTP1B (Fig 6D), and therefore the nectin1-independent spread pathway that functions in KO cells does not appear to involve either of these previously identified factors. Repeat siRNA knockdown of HVEM achieved a higher knockdown efficiency (Fig 6E), but virus spread was still maintained in these cells (Fig 6F, -AraC). Moreover, the same experiment carried out in the presence of AraC indicated that HVEM knockdown had little effect on the number of initially susceptible nectin1 KO cells (Fig 6F, +AraC), suggesting that HVEM was not a likely candidate for entry into this subpopulation of cells.

### Phenotype of HSV1 lacking glycoproteins gE or gI in the absence of nectin1

**Fig 7:**
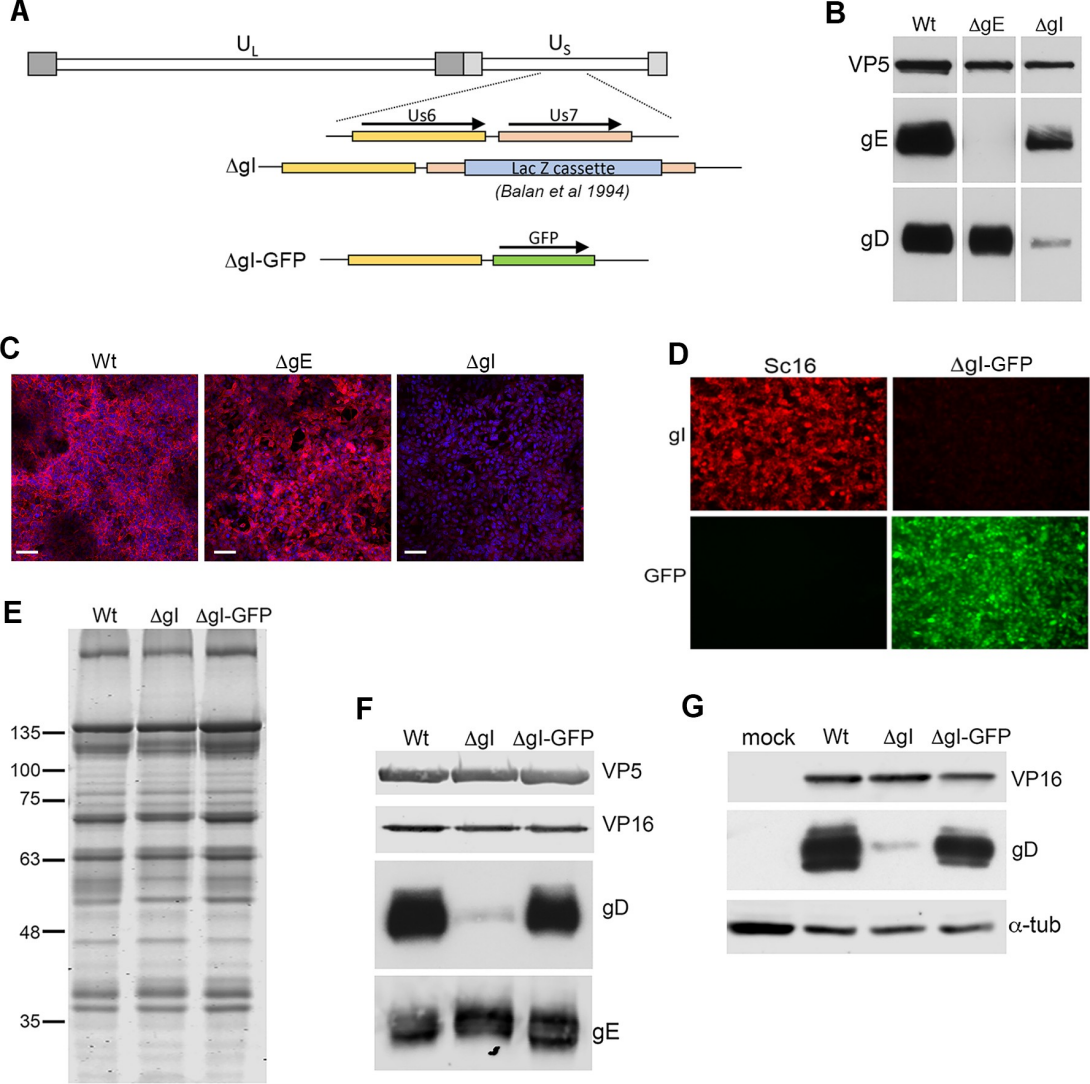
Differential gD expression in two independently generated gI deletion viruses. **(A)** Line drawing of the Sc16 ΔgI genome that was first described in [18], and the ΔgI-GFP constructed here by replacing the gI gene Us7 with the open reading frame for GFP using homologous recombination. The proximity of the gD-encoding gene Us6 is also indicated. **(B)** Extracellular virions were purified from HaCaT cells infected with Sc16 (Wt), ΔgE or ΔgI, and analysed by Western blotting for the major capsid protein VP5, and glycoproteins gE and gD. **(C)** nTERT cells infected with Sc16 ΔgE or ΔgI were fixed and permeabilised at 16 hours, and imaged for gI (stained in red) and nuclei stained with DAPI (blue). Scale bar = 100 μm. **(D)** Cells infected with Sc16 or ΔgI-GFP were fixed and permeabilised at 16 h, and imaged for gI (stained in red) and GFP (green). (**E)** and **(F)** Extracellular virions were purified from HaCaT cells infected with Sc16 (Wt), ΔgI or ΔgIGFP, and analysed by SDS-PAGE followed by staining with **(E)** Coomassie blue or **(F)** Western blotting for VP5, VP16, gD and gE. **(G)** nTERT cells infected with Wt, ΔgI or ΔgIGFP viruses were harvested at 16 h and analysed by SDS-PAGE and Western blotting for VP16, gD and α-tubulin.

HSV1 has two modes of spreading between cells: either by extracellular release and spread through the liquid medium to re-enter uninfected cells, or via direct cell-to-cell spread from already infected cells [15]. Infection by cell-to-cell transfer is known to involve glycoproteins gE and gI, which are both non-essential in tissue culture but essential *in vivo* [17,18], and viruses lacking either of these glycoproteins form small plaques [18]. Hence, we next tested the ability of HSV1 mutants in gE or gI, that had been constructed in an Sc16 background, to infect and spread in the nectin1 KO cells. While initiating these studies, we found that the ΔgI virus—previously constructed by insertion of the β-galactosidase gene into the gI-encoding Us7 open reading frame [18] (Fig 7A)—had an unusual phenotype which led us to examine its properties in more detail. Although it was confirmed to express no gI (Fig 7C), we also found that it incorporated a very low level of gD into extracellular virions compared to ΔgE or Wt virions (Fig 7B). We therefore constructed a further gI knockout virus in which Us7 was replaced by the GFP open reading frame (Fig 7A), confirming the lack of gI expression by immunofluorescence (Fig 7D). Wt, ΔgI and ΔgIGFP virions were purified from infected HaCaT cells and analysed by SDS-PAGE followed by Coomassie blue staining to equalise loading of virions, followed by Western blotting (Fig 7E and 7F). Strikingly, we found that while ΔgI virions contained a very low level of gD compared to Wt virions, ΔgIGFP virions contained roughly Wt levels of gD (Fig 7F). Moreover, Western blotting of infected cell lysates indicated that this low level of gD incorporated into the ΔgI virions correlated with a low level of gD expression, whereas gD expression was normal in the ΔgIGFP infected cells (Fig 7G). The original ΔgI virus–contained an insertion of β-galactosidase into the gI gene—therefore allowed us to assess virus spread in the presence of an unusually low level of the receptor binding protein gD.

**Fig 8:**
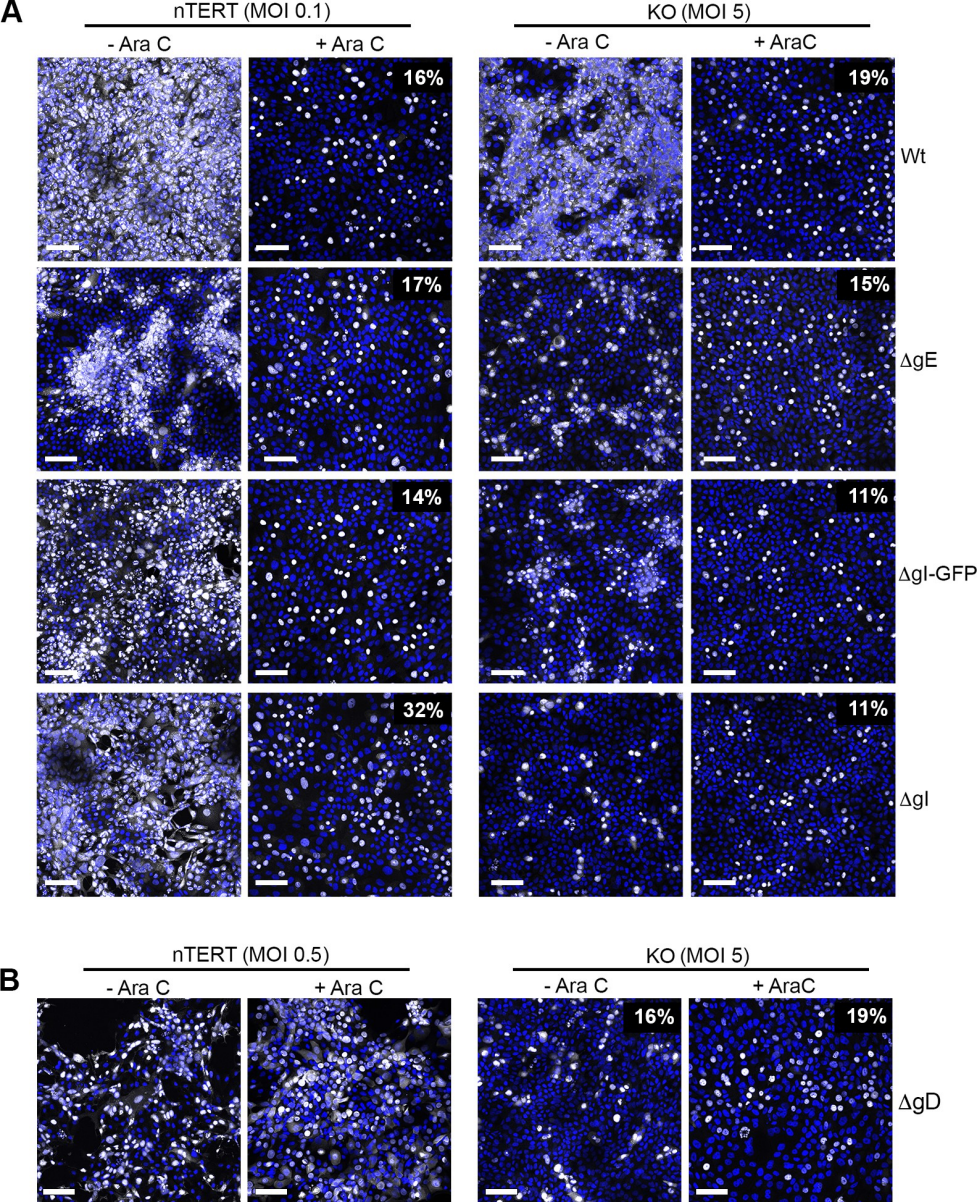
Spread phenotype of HSV1 lacking glycoproteins E, I or D in nectin1 KO cells. **(A)** nTERT and KO cells grown on coverslips were infected with HSV1 viruses as indicated at MOI 0.1 for nTERT and MOI 5 for KO cells in the absence or presence of 100 ng/ml AraC. Cells were fixed at 16 h, permeabilised and stained for ICP4 (white). **(B)** nTERT and KO cells grown on coverslips were infected with HSV1 lacking gD that had been propagated on a complementing gD cell-line, at MOI 0.5 for nTERT and MOI 5 for KO cells in the absence or presence of 100 ng/ml AraC for the course of the experiment. Cells were fixed at 16 h, permeabilised and stained for ICP4 (white). Nuclei were stained with DAPI (blue). Scale bar = 100 μm. Percentages refer to number of ICP4 positive nuclei, determined using NIH ImageJ software.

nTERT and KO cells were infected with each of these viruses in the absence or presence of AraC to determine their ability to infect the initial susceptible population of KO cells, and to establish if they can spread into neighbouring cells in a similar fashion to the Wt virus. Wt, ΔgE and ΔgIGFP viruses were all able to infect nTERT and KO cells with a similar efficiency, confirming that the absence of gE or gI had little effect on infection from outside in the presence or absence of nectin1 (Fig 8A, + AraC, compare percentages of ICP4 positive cells). Interestingly, the ΔgI virus which was shown above to contain a very low level of gD, infected relatively more nTERT cells than KO cells in comparison to the other viruses (Fig 8A, ΔgI +AraC). Infection in the absence of AraC further indicated that the glycoprotein mutant viruses were all able to spread in nTERT, although ΔgE appeared to spread less efficiently (Fig 8A, nTERT). Likewise, the ΔgE and ΔgIGFP viruses spread into surrounding KO cells, but at a reduced rate compared to Wt virus (Fig 8A, KO). By contrast, the ΔgI virus failed to spread into surrounding cells in the time-frame of this experiment suggesting its phenotype was different to that of ΔgIGFP virus (Fig 8A, KO). This suggests that the ΔgI virus is unable to spread in KO cells not just because of an absence of gI but because of its greatly reduced gD expression, and by extension that, not unexpectedly, gD is required for spread in nectin1 KO cells. As final confirmation, we utilised a gD knockout virus (ΔgD) that had been constructed in the same Sc16 background as the other glycoprotein mutants [25] and which had been propagated on gD-expressing Vero cells to produce a virus stock with a gD-positive phenotype but a gD-negative genotype. As expected, nTERT cells infected with this virus exhibited the same number of infected cells regardless of whether AraC was present or not, in line with the presence of gD in the virus inoculum but not the progeny virions (Fig 8B, nTERT). In agreement with the ΔgI results, the ΔgD virus was not able to spread in KO cells (Fig 8B, KO), confirming that the spread mechanism in the absence of nectin1 is still dependent on the major receptor binding protein, gD.

### Direct cell-to-cell transmission of HSV1 occurs in the absence of nectin1

**Fig 9:**
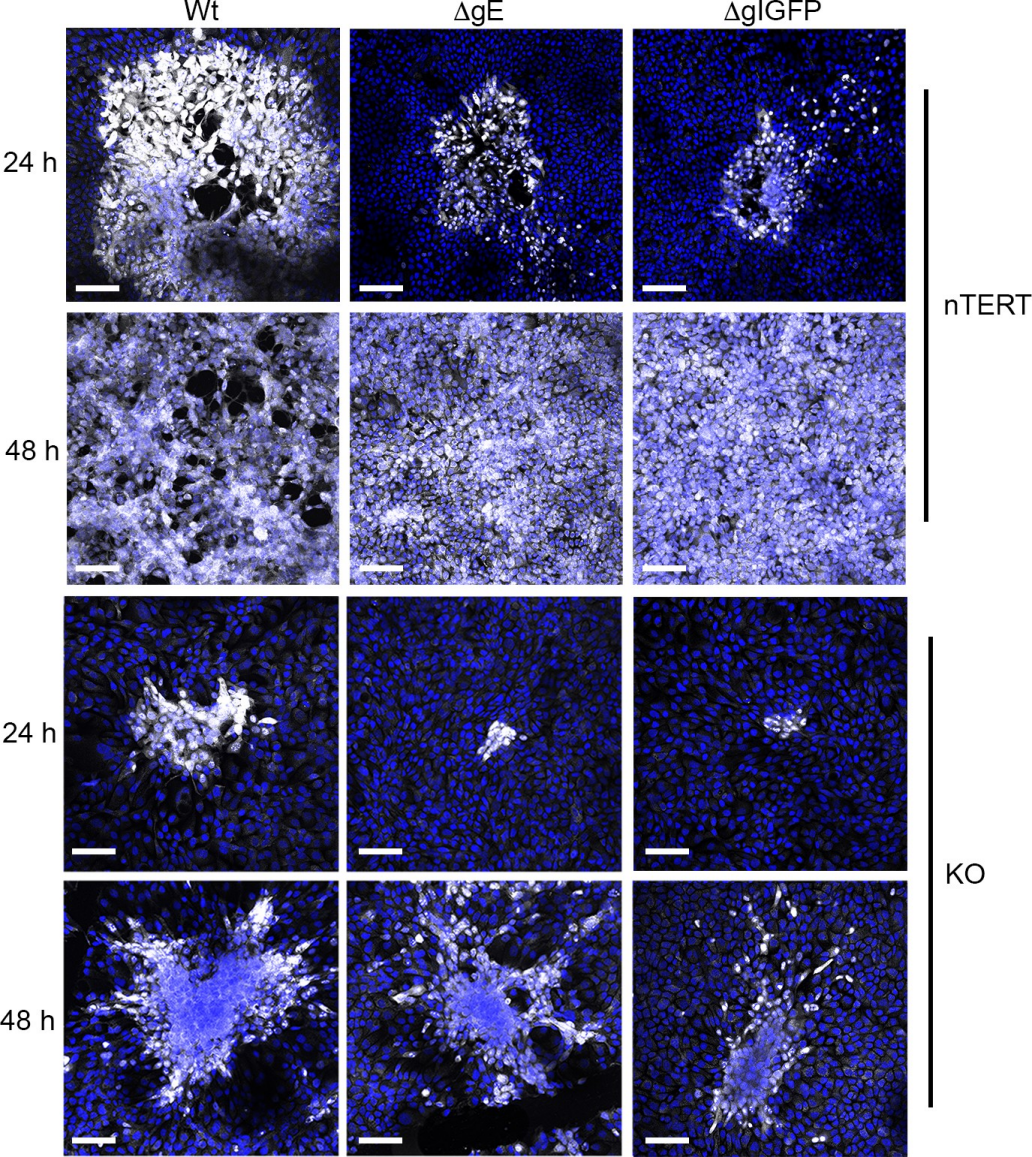
Relative spread of viruses in nectin1 KO cells. nTERT and KO cells grown on coverslips were infected with 50 or 2000 plaque forming units respectively of HSV1 Wt (Sc16), ΔgE or ΔgIGFP, fixed at 24 h or 48 h and stained for ICP4 (white). Nuclei were stained with DAPI (blue). Scale bar = 100 μm.

Extended spread of Wt, ΔgE and ΔgIGFP viruses was next determined by infecting nTERT and KO monolayers with a low number of virus plaque forming units, followed by fixing and staining for ICP4 at 24 h and 48 h (Fig 9). Wt foci were larger in nTERT cells compared to KO cells, indicating that although able to transmit, this process occurred more slowly in the absence of nectin1 (Fig 9). ΔgE and ΔgIGFP viruses were able to spread in nTERT cells but more slowly than Wt, as would be expected from their established phenotypes (Fig 9). Likewise, these viruses were able to spread over time in KO cells, but this was also at a slower rate than Wt virus (Fig 9).

**Fig 10:**
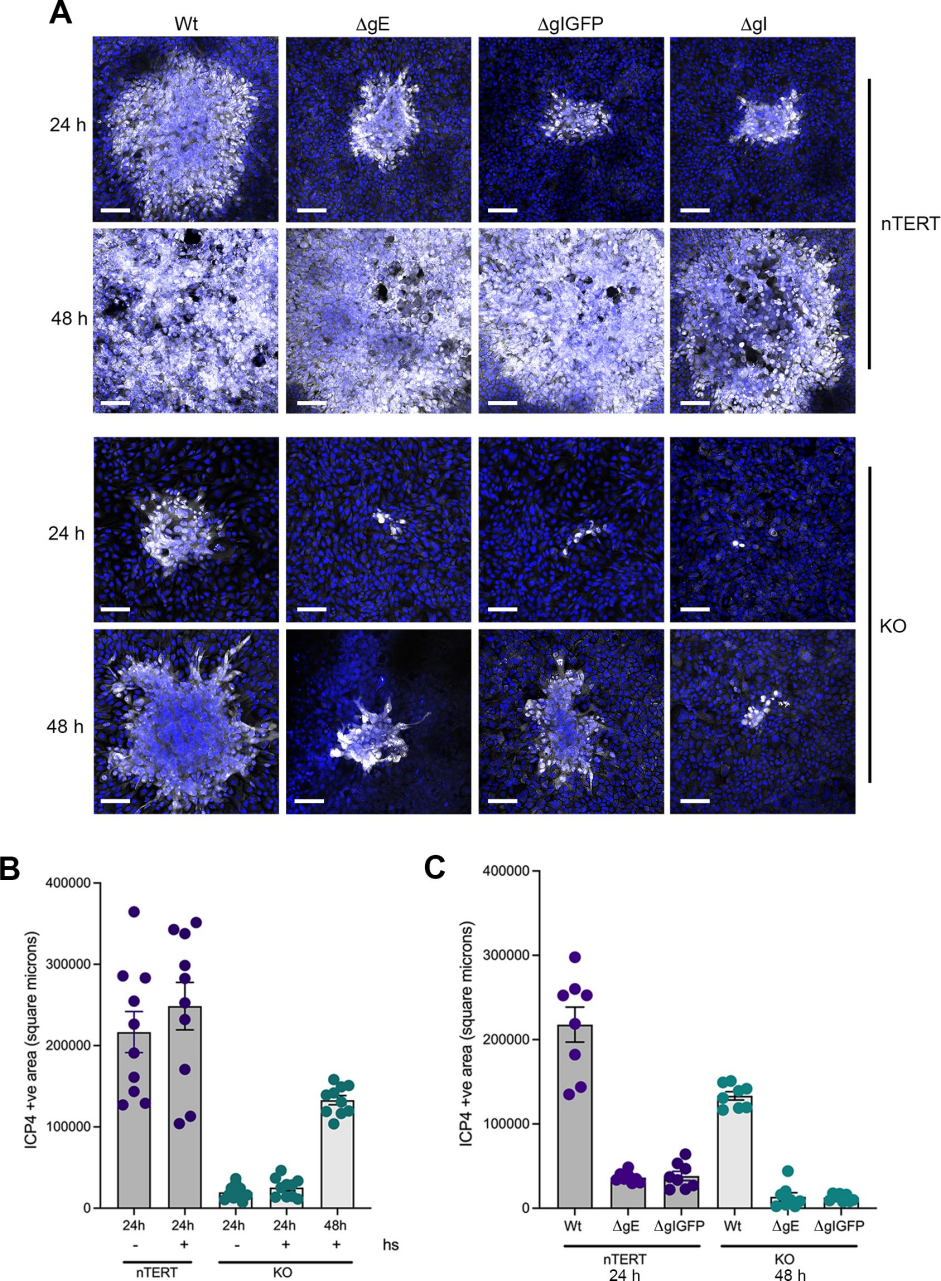
HSV1 spreads via cell-to-cell transmission in the absence of nectin1. **(A)** nTERT and KO cells grown on coverslips were infected with 50 or 2000 plaque forming units (calculated on Vero cells) respectively of HSV1 viruses as indicated in the presence of 10% human serum to neutralise extracellular virus and block extracellular spread. Cells were fixed at the indicated times, stained for ICP4 (white) and nuclei stained with DAPI (blue). Scale bar = 100 μm. **(B)** The area of ten ICP4-positive Wt foci on nTERT and KO cells infected in the absence or presence of human serum (hs) was measured using NIH ImageJ**. (C)** The area of eight ICP4-positive foci of Wt, ΔgE and ΔgIGFP viruses on nTERT and KO cells infected in the presence of human serum (hs) was measured using NIH ImageJ.

To confirm if HSV1 spreads cell-to-cell in nectin1 KO cells, low multiplicity infections of Wt, ΔgE, ΔgIGFP together with ΔgI were carried out in nTERT and KO cells in the presence of human serum, a well-utilised technique to neutralize extracellularly released virus, thereby eliminating extracellular spread and allowing virus spread only by direct transfer between cells in contact [15]. Initial studies indicated that 10% human serum was required to block the extracellular release of HSV1 from nTERT cells, as measured by comet-tail formation in plaque assays (S5 Fig). As in the absence of human serum, the glycoprotein deletion viruses were unable to spread to the same extent as Wt virus, but ΔgE, ΔgIGFP and ΔgI all spread similarly in nTERT cells (Fig 10A). In KO cells, this differential spread was similar for ΔgE and ΔgIGFP, but as seen above in Fig 8, the ΔgI virus all but failed to spread into surrounding cells (Fig 10A). Measurement of these infected cell foci confirmed that the inhibition of extracellular spread by human serum had no effect on the spread area of Wt infection in either nTERT or KO cells (Fig 10B), while the absence of gE or gI causes a similar relative reduction on foci size in nTERT and KO cells (Fig 10C). This confirms that HSV1 maintains the ability to spread in keratinocytes in the absence of nectin1, and that this spread occurs in a gE- gI-dependent fashion.

**Fig 11:**
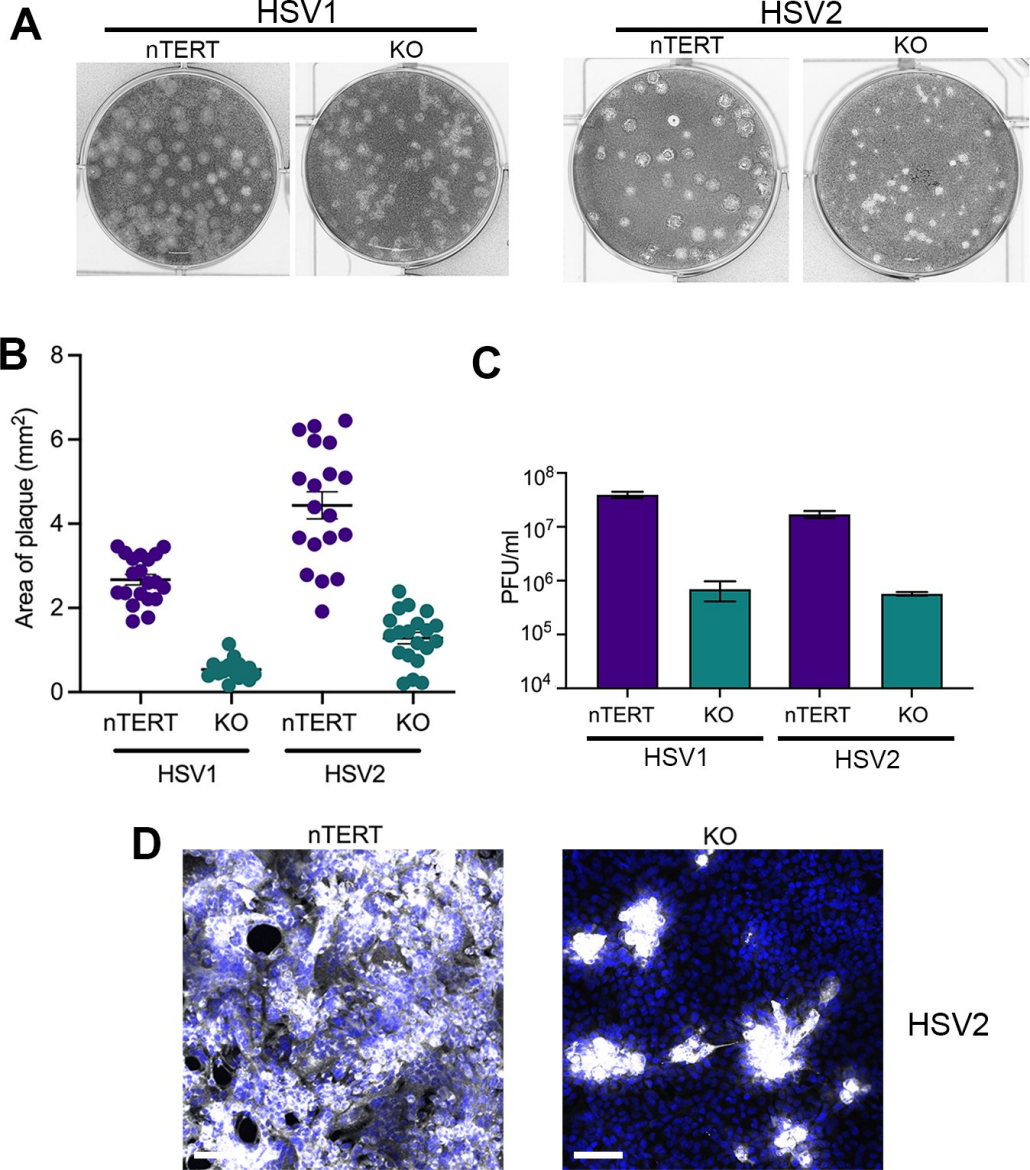
HSV types 1 and 2 form plaques on nectin1 KO cells. **(A)** HSV1 strain Sc16 and HSV2 strain HG52 was titrated onto nTERT and KO cells in the presence of 3% CMC, and fixed and stained with crystal violet 3 days later**. (B)** The area of ten HSV1 and HSV2 plaques on nTERT and KO cells shown in (A) was measured using NIH ImageJ**. (C)** The relative titre of HSV1 and HSV2 on nTERT and KO cells (mean±SEM, *n* = 3). **(D)** nTERT and KO cells were infected with HSV2 MOI 5 and fixed at 16 h. Cells were permeabilised and stained with an antibody to glycoprotein D (in white) and nuclei stained with DAPI (blue). Scale bar = 100 μm.

In line with the above results, titration of HSV1 on nTERT and nectin1 KO cells indicated that HSV1 was able to form plaques on nectin1 KO cells, although as expected from the foci staining experiments, these were smaller than plaques on nTERT cells (Fig 11A and 11B, HSV1). Moreover, the relative titre of HSV1 was around 50-fold lower in KO cells compared to nTERT cells (Fig 11C), reflecting the relative efficiency of initial infection seen above by flow cytometry and confocal microscopy. Interestingly, HSV2 behaved in the same way as HSV1 in all these assays, (Fig 11A, 11B and 11C, HSV2), while immunofluorescence of HSV2-infected monolayers confirmed the similarity between the two HSV types in the absence of nectin1 (Fig 11D). In all, this confirms that in spite of only being able to infect a small subpopulation of cells from the extracellular medium in the absence of nectin1, once in those cells, both HSV1 and HSV2 can go on to spread into the surrounding nectin1 KO cells which had been initially resistant to HSV1 entry. Furthermore, although nectin2 has been proposed as a receptor for HSV2 [11] and is well-expressed in these KO cells (Fig 1G), HSV2 cannot use nectin2 as a receptor in these human keratinocytes.

## Discussion

Like many viruses that infect host epithelia, HSV has two possible transmission routes: entry of cell-free particles from the extracellular environment, or direct movement between cells [16,17]. Despite the second route being important for spread in the host, the cell biology and molecular players involved in this mechanism are poorly understood. Here, we have not only proved conclusively that nectin1 is the major receptor for HSV1 to gain access from the extracellular environment in to human keratinocytes–the main cell type in the epithelia that HSV1 infects—but we have also demonstrated that cell-to-cell spread of HSV1 in these cells occurs in the absence of this receptor. Moreover, as anticipated from the original seminal report on the discovery of nectin1 as a prime candidate for an HSV entry receptor [7], HSV2 has the same entry requirements and spread properties as HSV1 in human keratinocytes. Consequently, once HSV has gained entry in to the small population of susceptible nectin1 KO cells, it is able to spread and form plaques by moving directly from cell-to-cell. This unexpected result shows for the first time that despite being the major receptor for entry from the extracellular environment, nectin1 is not required for HSV to move between keratinocyte cells in culture. Additionally, the second major HSV receptor known as HVEM [8] was also shown to be dispensable for cell-to-cell spread in the absence of nectin1 suggesting that this route of transmission occurs via a novel pathway which despite relying on gD does not require either of the two main gD receptors.

There are two possible means of intact virus particles undergoing cell-to-cell transfer. The first mechanism involves retention of the virion at the plasma membrane after egress from the infected cell, followed by binding to and rapid entry into the uninfected cell, similar to that seen in the HIV-induced T-cell virological synapse [26] or the projection of vaccinia virus particles into uninfected cells on actin tails generated from inside the infected cells [27]. Multiple ultrastructural studies have indicated that vast numbers of HSV1 virions line up between the plasma membranes of adjacent cells after egress from a range of cell types, indicating that external virions maintain interactions with the cell surface, providing a concentrated source of particles for potentially rapid transfer into uninfected cells. The second mechanism which we cannot exclude, is the transfer of virus infectivity between either pre-existing or virus-induced intercellular connections, formed between nectin1 KO cells. As such, this movement of infectivity could involve either fully assembled virions or virion sub-particles, for example in the form of unenveloped capsids moving between cell-to-cell contacts. Although results with the ΔUL34 virus confirmed that capsid export from the nucleus to the cytoplasm was required for the spread of infection, and imaging of GFP-tagged capsids showed capsid movement from infected into uninfected nectin1 KO cells, it is still to be formally proven that infectivity in these cells is transmitted in an enveloped virion rather than a naked capsid. Examples of such sub-particle transmission already exist in plant viruses, where genomes of, for example, tobacco mosaic virus are transferred between cells through plasmodesmata which have been dilated by the action of the virus movement protein [28]. Similar mechanisms of spread through intercellular connections known as tunnelling nanotubes have also been implicated in the spread of respiratory viruses including measles virus (MeV) and respiratory syncytial virus, which infect the simple columnar epithelia of the human respiratory tract [29,30]. In addition, MeV has also been shown to spread by cell contact in neurons [31]. Interestingly, there is recent evidence that the animal alphaherpesvirus bovine herpesvirus type1 may transmit through nanotubes in culture [32]. Transmission of HSV1 virions to nectin1-deficient CHO cells via microvesicles from infected oligodendrocytes has also been proposed as a novel route of virus spread [33].

Given the known role of the non-essential glycoproteins gE and gI in cell-to-cell transfer in epithelial and neuronal cells [17,34–36], it is maybe not surprising that the nectin1 independent cell-to-cell spread mechanism of HSV1 was shown here to involve these glycoproteins. Although the functions of gI and gE have not been fully defined, they are essential *in vivo* [17,18], and are therefore candidate factors to facilitate the transfer of virions from one keratinocyte to another. Indeed, they have previously been shown to be involved in the sorting of nascent virions to cell junctions in polarised epithelial cells [37]. In addition, they are required for anterograde transport of virions in neuronal axons [38,39], by recruiting specific kinesins for microtubule movement [40,41], a mechanism that could also function in epithelial cells. However, ΔgI and ΔgE virions are efficiently released from epithelial cells in culture suggesting that transport to the plasma membrane is unhindered in these cells [17,34], and it is therefore unlikely that this kinesin-mediated microtubule transport role is generally applicable for delivery to the plasma membrane of epithelial cells. Nonetheless, this mechanism may channel virions for delivery on microtubule tracks to the specific areas of the plasma membrane that form cell-to-cell contacts, thereby potentially delivering them to the correct site for rapid spread. Moreover, it was recently suggested that inhibition of the ER-bound tyrosine phosphatase, PTP1B, specifically blocked cell-to-cell spread of HSV1, suggesting that tyrosine phosphorylation regulates either the cellular or viral trafficking proteins that make up the cell-to-cell spread machinery [20]. However, those experiments were carried out in HaCaT cells, and in our hands PTP1B was not required for cell-to-cell spread as shown by siRNA depletion here. Future live cell and ultrastructural studies of the nectin1 KO keratinocytes will help to delineate how these different features integrate to facilitate cell-to-cell spread of HSV1 and how the proteins of the virus spread machinery including gI and gE, contribute to this process.

Cell-to-cell spread of virus in the host allows the virus to avoid neutralizing antibodies and some intrinsic/innate antiviral responses whilst providing an efficient and rapid way for small numbers of virus particles or sub-particles to disseminate infection. It is of note then that clinical isolates of HSV1 have the propensity to be cell-associated when first isolated, whereas lab strains often produce large amounts of free virus [15]. This is similar to the situation in human cytomegalovirus, a betaherpesvirus that is predominantly cell-associated in the host, but rapidly loses a large part of its genome over as few as three passages in cultured fibroblasts, resulting in virus that is less cell-associated and reduced in pathogenicity [42]. Moreover, varicella zoster virus (VZV), another human alphaherpesvirus which infects keratinocytes in the host to cause chickenpox or shingles, is highly cell- associated in culture [43], and unlike other alphaherpesviruses which can spread by the extracellular route, VZV does not have a homologue of HSV1 gD for receptor binding from the outside [43]. Taken together, this indicates that the cell-to-cell spread machinery is vital for VZV infection and as such, it is intriguing to speculate that our nectin1 KO keratinocytes may recapitulate the natural spread mechanism of VZV but in an HSV background.

There is growing evidence that human keratinocytes express a much higher level of nectin1 than many other human cell-types used for HSV1 studies [13]. Nonetheless, although the vast majority of nectin1 KO cells were found here to be resistant to HSV1 entry from outside, a small proportion remained susceptible, indicating entry via an alternative, as yet unidentified receptor. The number of cells infected could reflect a requirement for the cells to be, for example at a certain stage of the cell-cycle for virus entry to occur in the absence of nectin1. However, the fact that prolonged exposure to the virus inoculum did not result in an increased number of initially infected cells suggests this is unlikely to be the case. Alternatively, another receptor may be expressed at the cell surface in this small population of cells allowing virus binding and entry into those cells. Depletion of the alternative gD receptor HVEM in the nectin1 KO cells had little effect on virus entry in to the susceptible KO cells, and although we can not rule out that a residual amount of HVEM is enough to facilitate entry (or spread), other candidate HSV1 receptors such as 3-O-sulfated heparin sulfate [10] or the gB receptor PILRα [12] will need to be investigated. Interestingly, nectin2 has previously been shown to act as a receptor for HSV2 but not HSV1 [11] and yet does not seem to function for HSV2 entry into human keratinocytes in the absence of nectin1 (see Fig 11). It is also possible that the reduced susceptibility is virus-related and not cell-related, with only a sub-population of virions competent for entry into nectin1 KO cells. This would be due to the specific complement of proteins present in those virions meaning that the entry receptor could well be present on all cells, but that only 5 to 10% of the virion population would be competent for receptor binding. Whatever the mechanism and receptor involved, it is clear that these pathways of entry are minor components of the entry process, and that the expression of nectin1 at the keratinocyte cell surface facilitates the efficient entry of HSV types 1 and 2 from the extracellular environment.

The results presented here imply that HSV1 requires nectin1 for host-to-host transmission, facilitating virus entry into the host epithelium. However, there is a real possibility that once the virus has gained access into its first target cell, nectin1 is not required in virus spread at least within the epithelium. It is noteworthy that HSV1 infects multi-layered, stratified epithelia, where individual keratinocytes form cell-to-cell contacts in all dimensions. Work is now needed on three-dimensional raft cultures of keratinocytes to determine if our results in keratinocyte monolayers translate to a model tissue and ultimately the host. Our nectin1 KO cells–and in particular the ease with which we can image GFP-tagged capsid movement in these cells–provide new scope to study the spread of HSV types 1 and 2 in all dimensions in the absence of confounding effects from released extracellular virus.

## Materials and methods

### Cells and viruses

nTERT cells were cultured in 3:1 Dulbecco’s modified Eagle media (DMEM) to Hams F12 media supplemented with RM+ supplement (10 ng/ml mouse epidermal growth factor (Serotec), 1 ng/ml cholera toxin (Sigma), 400 ng/ml hydrocortisone (Sigma), 5 μg/ml apo-transferrin (Sigma) and 13 ng/ml liothyronine (Sigma), 50 U/ml penicillin streptomycin and 10% foetal bovine serum (FBS)). Vero, HeLa and HaCaT cells were cultured in DMEM supplemented with 50 U/mL penicillin/streptomycin and 10% FBS. Vero cells constitutively expressing gD (VD60) have been described before [25]. All HSV viruses except ΔUL34 and ΔgD were routinely propagated in Vero cells in DMEM supplemented with 50 U/mL penicillin/streptomycin and 2% FBS. Infections were carried out by adding inoculum in DMEM plus 2% FBS for one hour at 37 ^o^C, unless otherwise stated. Inoculum was removed, cells were washed in DMEM and replaced with DMEM plus 2% FBS, and left at 37 ^o^C for the rest of the experiment. Plaque assays were carried out in nTERT in RM+ media supplemented with 2% FBS, 50 U/mL penicillin/streptomycin with 3% carboxymethyl cellulose (CMC) as an overlay. Cell-to-cell spread assays were carried out using pooled human serum (BioIVT) at a concentration of 10% to neutralise extracellular virus. Arabinofuranosyl Cytidine (AraC) was used at a concentration of 100 ng/ml in media to inhibit HSV1 genome replication. VACV was propagated in RK13 cells and titrated on BSc1 cells.

HSV1 strain Sc16 was routinely used in this study [44]. The HG52 strain of HSV2 was used here. Strain s17 expressing GFP-VP22 or GFP-VP26, and Sc16110lacZ have been described previously [21,45,46]. The ΔgD, ΔgI and ΔgE viruses have been described before [18,25]. Sc16 expressing GFP in place of Us7 (ΔgIGFP) was constructed using homologous recombination of Sc16 genomic DNA with a plasmid containing the GFP gene surrounded by the Us7 flanking regions, followed selection of GFP-positive plaques and a further three rounds of plaque purification (Fig 7A). VACV western reserve (WR) strain expressing A5-GFP was a gift from Carlos Maluquer de Motes (University of Surrey) and has been described before [47]. HSV1 deleted for UL34 and UL34-expressing Vero cells for its propagation [24] were a gift from Richard Roller (University of Iowa). Extracellular virions were prepared as described previously [48]. Briefly, approximately 6 x 10^8^ HaCaT cells were infected at a multiplicity of 0.02, and the extracellular medium was collected and centrifuged at 3,000 rpm once cytopathic effect was advanced (3 to 4 days post-infection) to remove cell debris. Virus particles were then pelleted from the cleared supernatant at 9,000 rpm, the pellet was resuspended phosphate buffered saline (PBS) and carefully layered onto a preformed 5% to 15% (wt/vol) Ficoll gradient. Gradients were centrifuged at 12,000 rpm for 2 h at 4 ^o^C in an SW41 Ti swinging-bucket rotor. Virions were harvested by needle puncture through the side of the tube, diluted in 10 ml PBS, and pelleted at 25,000 rpm for 1 h. The pellets were resuspended in PBS and stored at -80 ^o^C.

### Antibodies used in this study

The following primary antibodies were kindly provided by: Colin Crump (University of Cambridge), mouse anti-gD (LP14) and mouse anti-VP16 (LP1); David Johnson (Oregon health and Science University), mouse anti-gI (3104); Carlos Maluquer de Motes (University of Surrey), mouse anti-F13. Commercially available antibodies were as follows: mouse anti-ICP0 (11060; Santa Cruz); mouse anti-ICP4 (Santa Cruz); mouse anti-nectin1 (R1.302; Biolegend); mouse anti-nectin2 (TX31; Biolegend); mouse anti-α tubulin (Sigma).

### SDS-PAGE and Western blotting

Samples were separated on SDS-PAGE gels from 8–14% as appropriate and transferred to nitrocellulose membranes before incubation with primary antibody. Goat anti-mouse IRDye 680RD and goat anti-rabbit IRDye 800CW (LI-COR Biosciences) secondary antibodies were used as appropriate, before blots were either imaged using an Odyssey CLx imaging system (LI-COR Biosciences) or developed using SuperSignal West Pico chemiluminescent substrate and exposed to X-ray film.

### Flow cytometry

Cell surface staining for nectin1 was performed on live cells that had been blocked by incubation in PBS with 5% FBS and 1 mM EDTA for 10 min. Mouse anti-nectin1 was added in PBS with 5% FBS and 1 mM EDTA for 30 min. After extensive washing with PBS, Alexafluor 488 anti-mouse secondary antibody was added in PBS with 5% FBS and 1 mM EDTA and incubated for a further 30 min. Cells were then washed before being fixed in 1% PFA for 20 min, suspended in PBS with 1% FBS and 1 mM EDTA and analysed using a BD FACS Celesta flow cytometer. Infected cells were trypsinised and fixed as above then directly analysed for GFP fluorescence. Results were analysed using FlowJo software.

### Inhibition of virus entry using nectin1 antibody

One-day-old confluent monolayers of cells grown on coverslips were prechilled on ice for 30 mins. Culture medium was removed and replaced with 10 μg of nectin1 or nectin2 antibody diluted in ice-cold infection medium, and cells were incubated for 1 hr before addition of HSV1 GFP22 for a further 30 mins on ice. Cells were then incubated at 37 ^o^C for 15 mins before being subjected to acid-washing to inactivate unpenetrated virus. Cells were returned to 37 ^o^C, fixed after 4 hr incubation, mounted in Mowiol containing DAPI, and GFP positive cells detected by confocal microscopy.

### CRISPR-Cas9 knockout of nectin1

Two guide RNA sequences targeting nectin-1 (GACTCCATGTATGGCTTCATCGG and GAGTCGTTCACCTGGACCACCTGG) were inserted as annealed oligonucleotides with BBS1 overhangs into BBS1 digested plasmid pX459 which expresses Cas9. Plasmids were transfected into nTERT cells in a 1:1 ratio with Lipofectamine 2000 (Invitrogen). For KO cells media was replaced 24 hours later with media containing 500 ng/μl puromycin and incubated for a further 72 h. Surviving cells were trypsinised to a single cell suspension, added to 96 well plates at a concentration of 5 cells/ml and grown as clonal populations. For KO2 cells, cells were trypsinised to a single cell suspension 24 h after transfection. Live cells were blocked and stained as for flow cytometry above, and fluorescence associated cell sorting (FACS) was performed using a FACS ARIA fusion (BD Biosciences). Nectin1 negative cells were identified by gating using FACS DIVA v8 software and single cell sorted into 96 well plates containing filtered conditioned media. Once confluent potential clones were expanded and screened for nectin1 deletion.

### Construction of keratinocytes expressing epitope-tagged nectin1

Plasmid pNectinV5 [49] was transfected into KO2 cells with Lipofectamine 2000. Seventy-two hours post transfection cells were moved to a 33°C incubator for 5 days to promote homologous recombination. Live cells were blocked and nectin1 stained for flow cytometry as above. Nectin1 positive cells were then sorted into 96 well plates containing filtered conditioned media. Once confluent, potential clones were screened for V5 expression.

### Genomic sequencing

Genomic DNA was extracted from cells using the DNeasy blood and tissue kit (Qiagen). Nectin1 deletion was confirmed by PCR amplification of the 5’ region of the *NECTIN1* gene (Forward and reverse primers: GACTCAGCTGCGAGGGAGAAG and GAGCTGGCTTTCTCGATTGCC)., Amplicons were confirmed to be the correct size, purified and expanded in a Topo cloning vector (Invitrogen). Six KO and three KO2 clones were purified and sent for Sanger sequencing (Eurofins Genomics).

### Transfection of siRNA

siRNAs to HVEM and PTP1B (Ambion, ThermoFisher Scientific) were reverse transfected with Lipofectamine 2000 to a final concentration of 20 nM and left for 48 h. The Silencer Select negative control siRNA number 1 was used as a negative control (Ambion, ThermoFisher Scientific).

### Quantitation of mRNA by RT-qPCR

Total RNA was isolated using an RNeasy mini kit (Qiagen) then DNase I (Invitrogen) treated according to the manufacturers protocol. cDNA synthesis was performed with Superscript III (Invitrogen) and random hexamers according to the manufacturer’s instructions. Quantitative polymerase chain reaction (qPCR) was performed with the MESA BLUE qPCR kit for SYBR assay (Eurogentec) on a LightCycler96 system (Roche) using primers shown in S1 Table.

### Immunofluorescence and confocal microscopy

Cells for immunofluorescence were grown on coverslips and fixed with 4% paraformaldehyde in PBS for 20 min at room temperature, followed by permeabilisation with 0.5% Triton-X100 for 10 min. Fixed cells were blocked by incubation in PBS with 10% NCS for 20 min, before the addition of primary antibody in PBS with 10% NCS, and a further 30-min incubation. After extensive washing with PBS, the appropriate Alexafluor conjugated secondary antibody was added in PBS with 10% NCS and incubated for a further 30 min. The coverslips were washed extensively in PBS and mounted in Mowiol containing DAPI. Images were acquired using a Nikon A1 confocal microscope and processed using ImageJ software [50].

## Supporting information

S1 Table qPCR primers used for measuring siRNA knockdown efficiency. (TIF)

S1 Fig Nucleotide sequence of nectin1 surrounding gRNA target site in CRISPR-Cas9 edited nTERT cells. KO#1 and KO#2 are the two sequences found in the KO line, KO2 is the single sequence found in KO2.
(TIF)

S2 Fig Characterisation of Cas9 expression in nectin1 knockout cell lines. Cell lysates from parental nTERT and nectin1 KO and KO2 lines were subjected to SDS-PAGE and Western blotting for Cas9 and α-tubulin as a loading control.
(TIF)

S3 Fig Expression of V5-tagged nectin1 by transient transfection. **(A)** nTERT and HeLa cells were transfected with plasmid expressing nectin1V5, harvested at 16 h and analysed by SDS-PAGE and Western blotting for V5. **(B)** As for (**A**), but nectin1V5 transfected nTERT cells were harvested and lysates subjected to deglycosylation with PNGaseF prior to analysing by SDS-PAGE and Western blotting. (**C**) nTERT cells grown on coverslips were transfected with nectin1V5-expressing plasmid. Sixteen hours later, cells were either cell-surface stained with antibody to the extracellular domain of nectin1 prior to fixation, or fixed and permeabilised followed by staining with the same antibody (green). Nuclei were stained with DAPI (blue). Scale bar = 20 μm.
(TIF)

S4 Fig Time-course of HSV1 transmission in nTERT cells. Confluent nTERT cells were infected with Sc16 at MOI 0.01 in the absence or presence of 100 ng/ml AraC, fixed and permeabilised at the indicated times and stained for ICP4 (white) and nuclei were stained with DAPI (blue). Scale bar = 100 μm.
(TIF)

S5 Fig Inhibition of extracellular spread by increasing concentration of human serum. Confluent nTERT cells were infected with approximately 10 pfu of Sc16 and incubated for 3 days in media containing the indicated concentration of human serum. Increasing levels of human serum inhibit the appearance of comet tails which are a consequence of extracellular virus spread.
(TIF)
