# Supplementary figures and images for: Cell-to-cell transmission of HSV1 in human keratinocytes in the absence of the major entry receptor, nectin1
Source: PLoS Pathog. 2021 Sep 29;17(9):e1009631. doi: 10.1371/journal.ppat.1009631 (PMC8505007; doi:10.1371/journal.ppat.1009631)

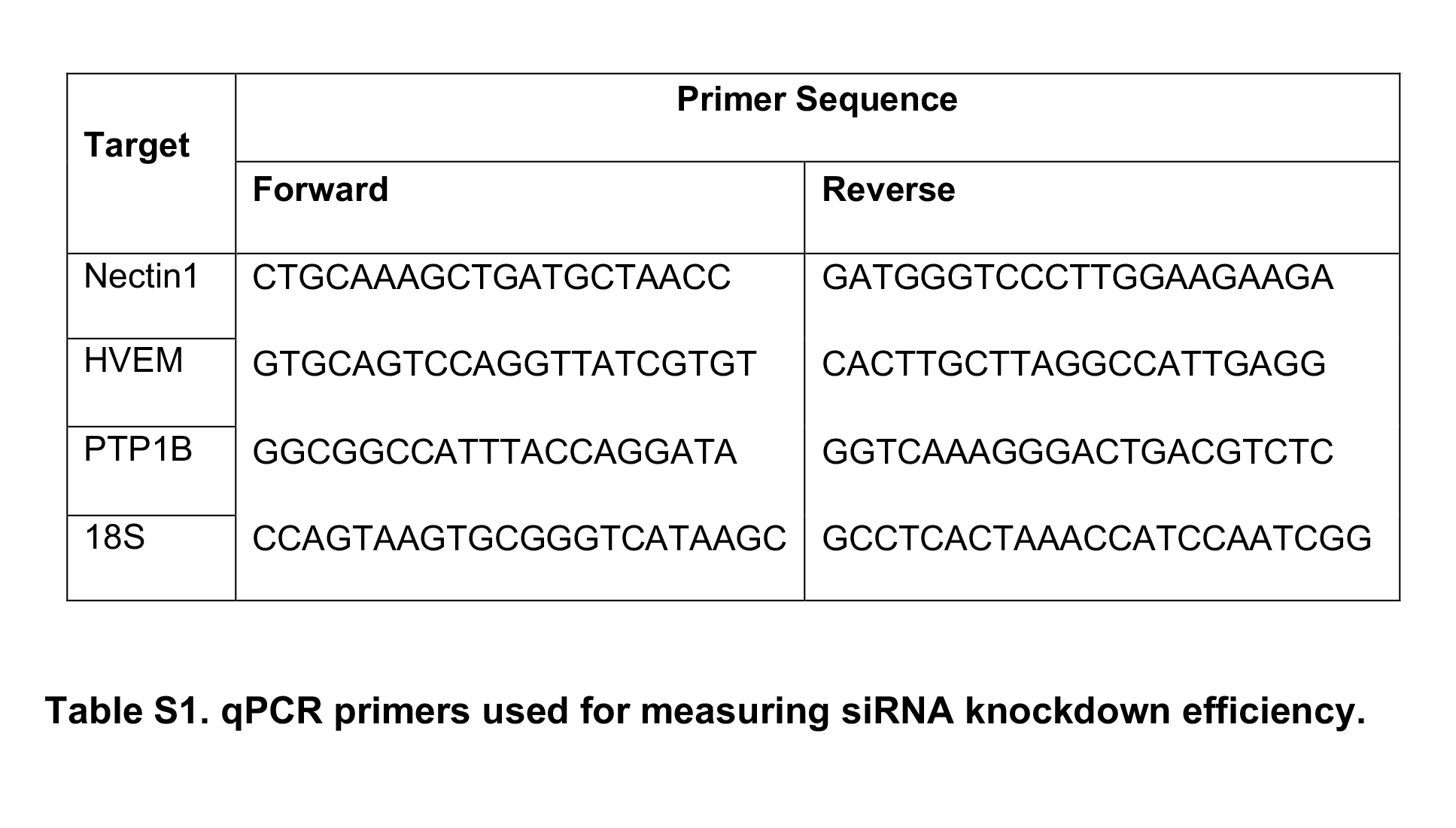

Supplement: S1 Table — (TIF) [file ppat.1009631.s001.tif]

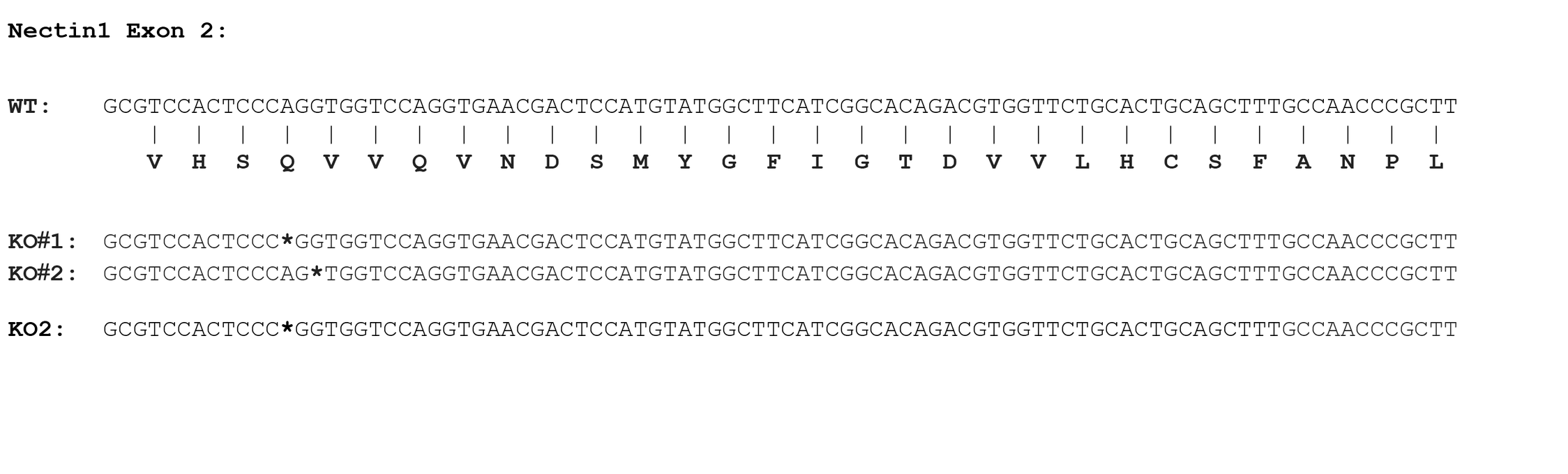

Supplement: S1 Fig — KO#1 and KO#2 are the two sequences found in the KO line, KO2 is the single sequence found in KO2. (TIF) [file ppat.1009631.s002.tif]

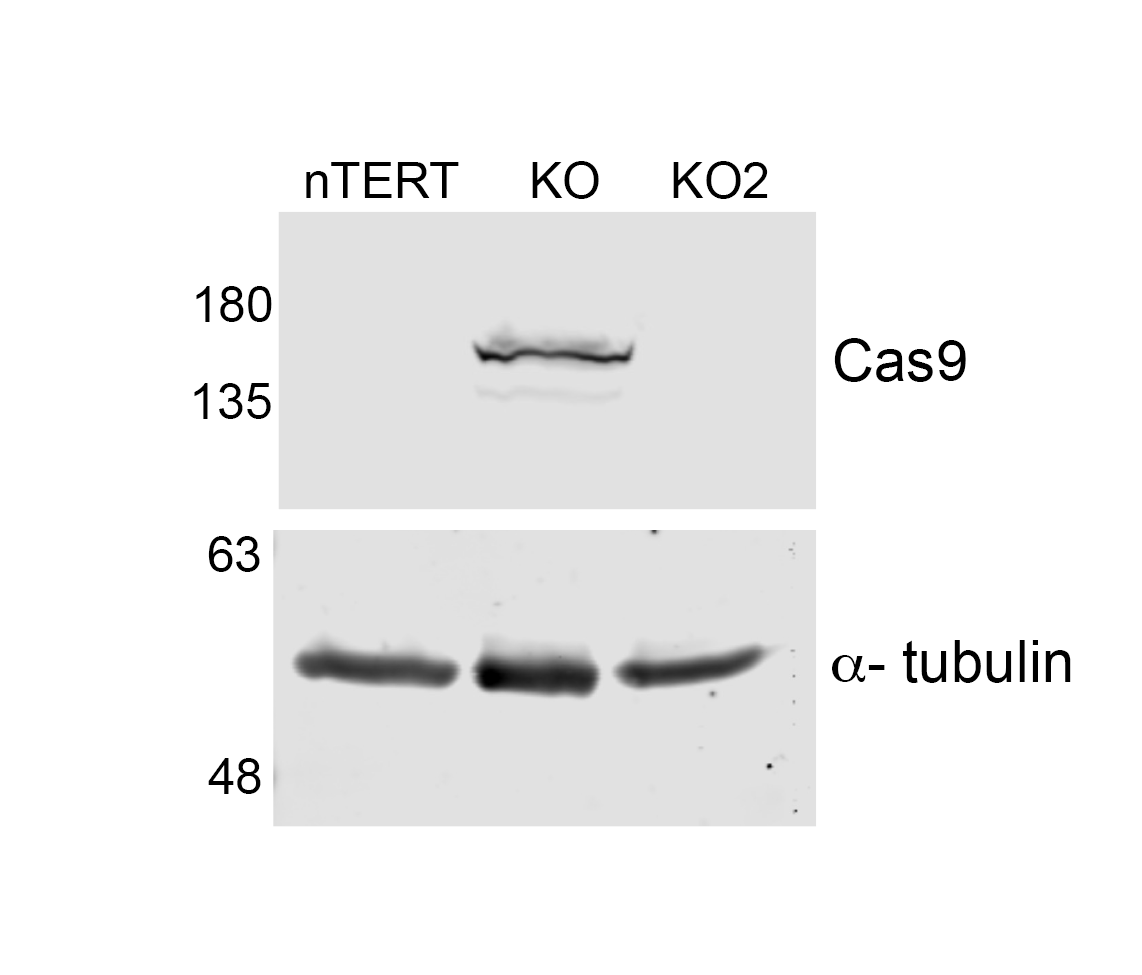

Supplement: S2 Fig — Cell lysates from parental nTERT and nectin1 KO and KO2 lines were subjected to SDS-PAGE and Western blotting for Cas9 and α-tubulin as a loading control. (TIF) [file ppat.1009631.s003.tif]

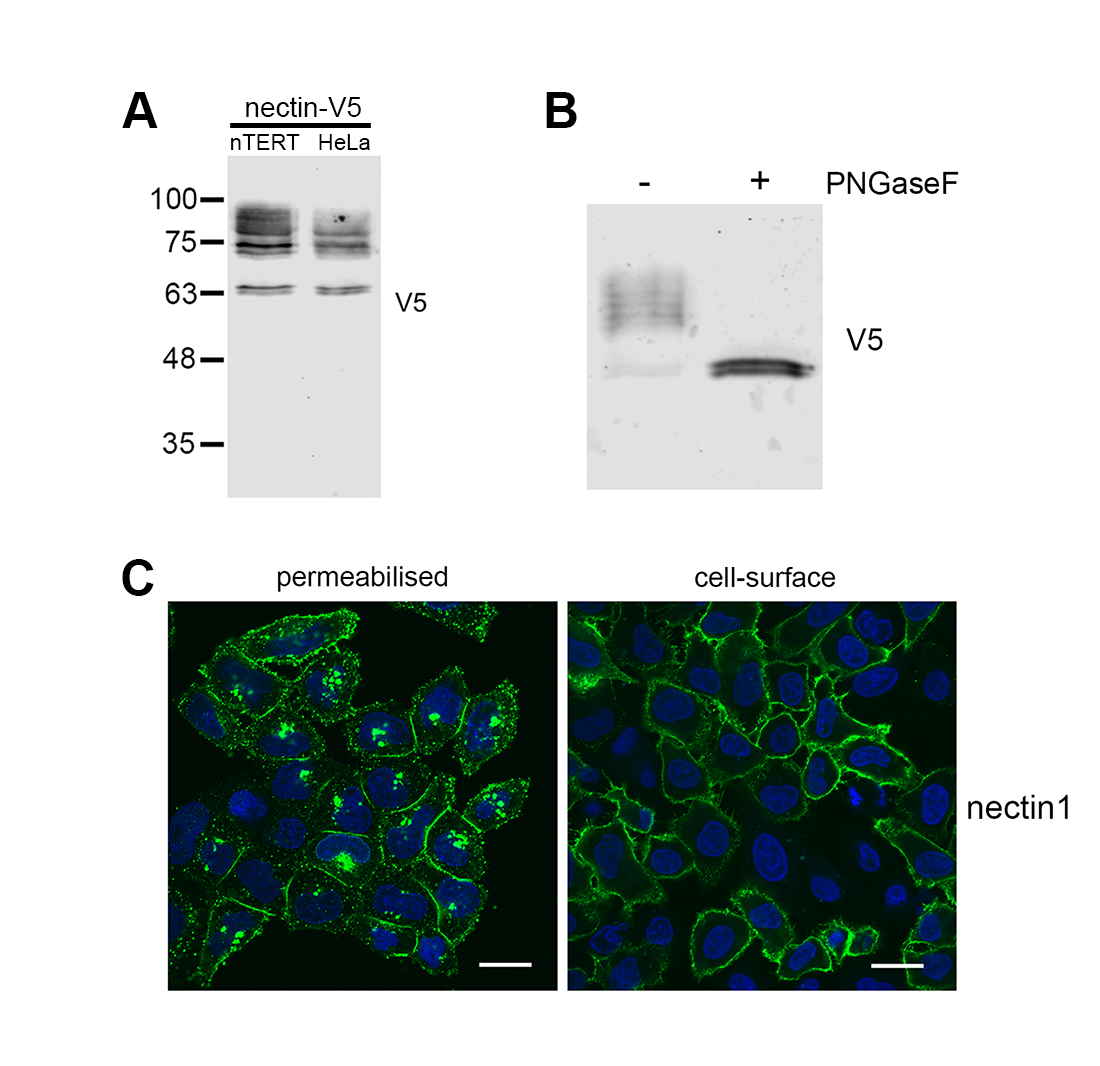

Supplement: S3 Fig — (A) nTERT and HeLa cells were transfected with plasmid expressing nectin1V5, harvested at 16 h and analysed by SDS-PAGE and Western blotting for V5. (B) As for (A), but nectin1V5 transfected nTERT cells were harvested and lysates subjected to deglycosylation with PNGaseF prior to analysing by SDS-PAGE and Western blotting. (C) nTERT cells grown on coverslips were transfected with nectin1V5-expressing plasmid. Sixteen hours later, cells were either cell-surface stained with antibody to the extracellular domain of nectin1 prior to fixation, or fixed and permeabilised followed by staining with the same antibody (green). Nuclei were stained with DAPI (blue). Scale bar = 20 μm. (TIF) [file ppat.1009631.s004.tif]

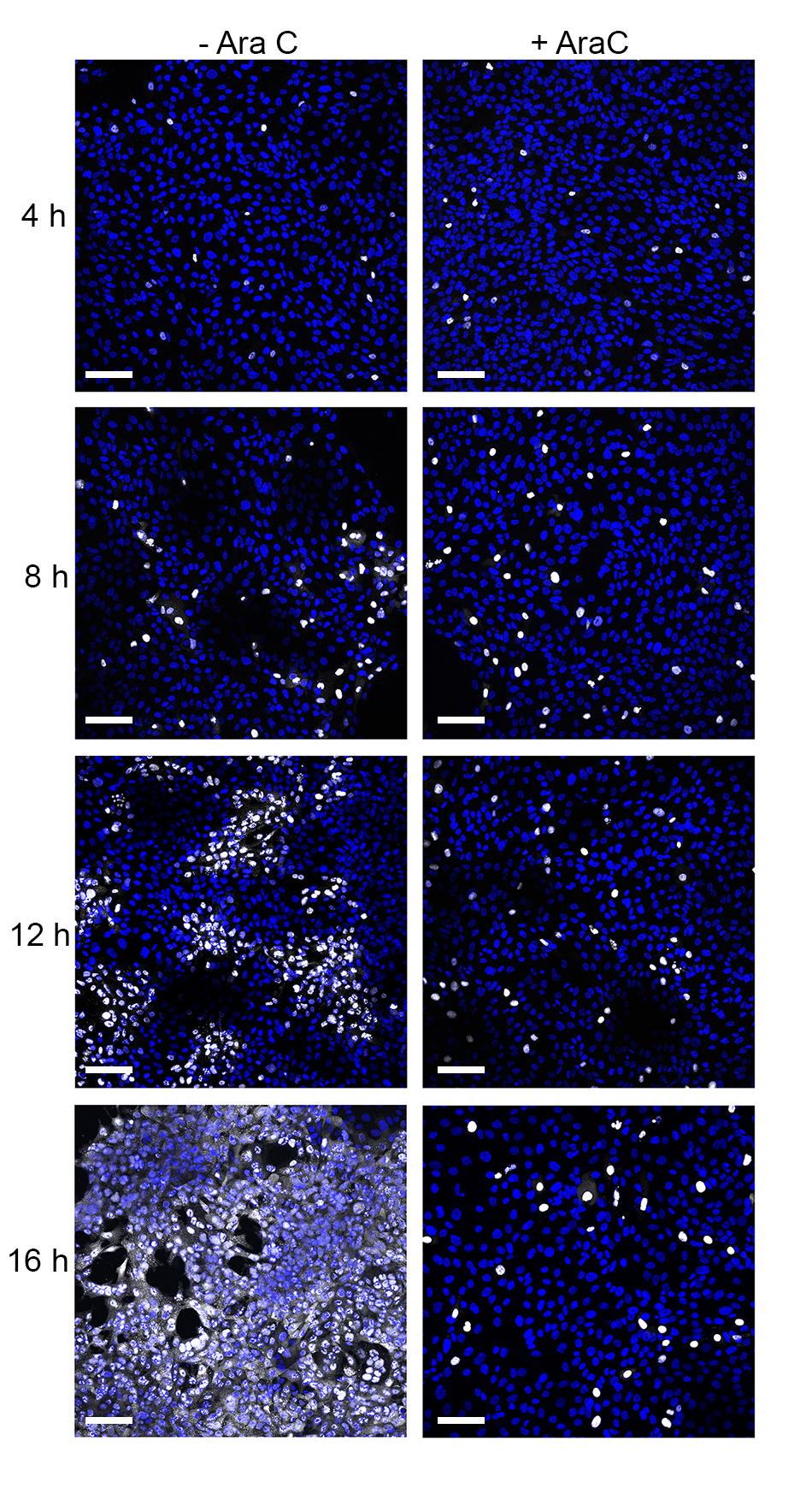

Supplement: S4 Fig — Confluent nTERT cells were infected with Sc16 at MOI 0.01 in the absence or presence of 100 ng/ml AraC, fixed and permeabilised at the indicated times and stained for ICP4 (white) and nuclei were stained with DAPI (blue). Scale bar = 100 μm. (TIF) [file ppat.1009631.s005.tif]

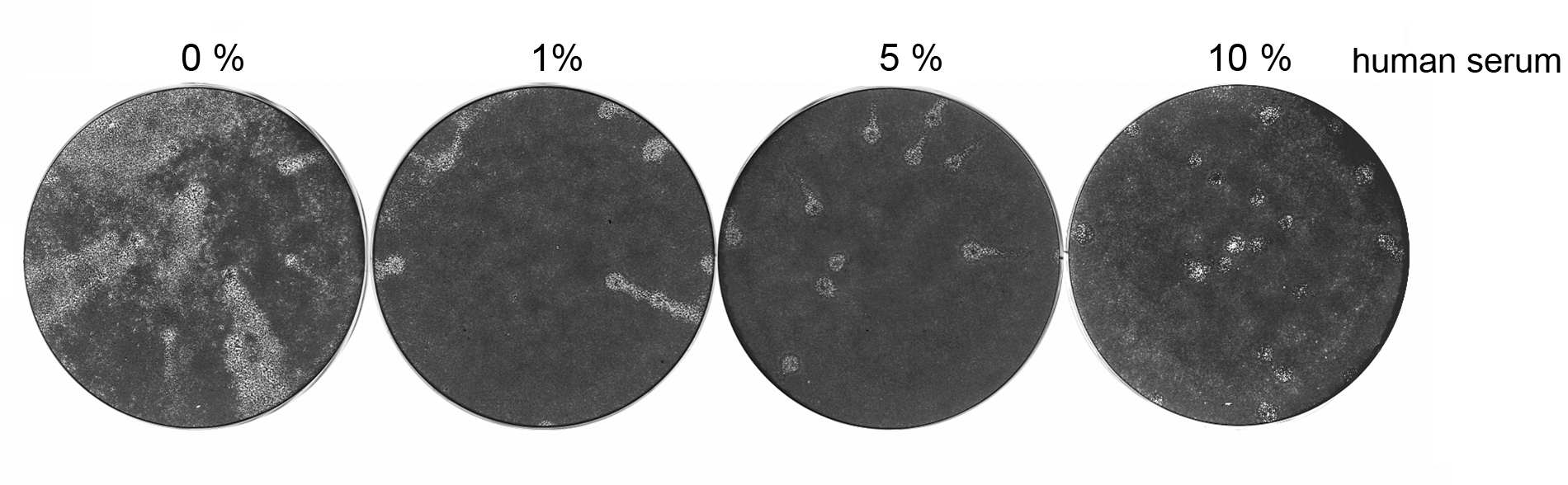

Supplement: S5 Fig — Confluent nTERT cells were infected with approximately 10 pfu of Sc16 and incubated for 3 days in media containing the indicated concentration of human serum. Increasing levels of human serum inhibit the appearance of comet tails which are a consequence of extracellular virus spread. (TIF) [file ppat.1009631.s006.tif]
